# RNA-mediated paternal heredity of diet-induced obesity and metabolic disorders

**DOI:** 10.1038/srep18193

**Published:** 2015-12-14

**Authors:** Valérie Grandjean, Sandra Fourré, Diana Andrea Fernandes De Abreu, Marie-Alix Derieppe, Jean-Jacques Remy, Minoo Rassoulzadegan

**Affiliations:** 1 Inserm, U1091, Nice, F-06108; 2 CNRS, UMR7277, F-06108, France; 3 University of Nice-Sophia Antipolis, UFR Sciences, Nice, F-06108; 4 Institut de Pharmacologie Moléculaire et Cellulaire (IPMC), UMR 6079 CNRS-UNSA, Sophia Antipolis, France; 5 UMR Inra 1355, CNRS 7254, UNS, Sophia Antipolis, France

## Abstract

The paternal heredity of obesity and diabetes induced by a high-fat and/or high-sugar diet (Western-like diet) has been demonstrated through epidemiological analysis of human cohorts and experimental analysis, but the nature of the hereditary vector inducing this newly acquired phenotype is not yet well defined. Here, we show that microinjection of either testis or sperm RNA of male mice fed a Western-like diet into naive one-cell embryos leads to the establishment of the Western-like diet-induced metabolic phenotype in the resulting progenies, whereas RNAs prepared from healthy controls did not. Among multiple sequence differences between the testis transcriptomes of the sick and healthy fathers, we noted that several microRNAs had increased expression, which was of interest because this class of noncoding RNA is known to be involved in epigenetic control of gene expression. When microinjected into naive one-cell embryos, one of these small RNA, i.e., the microRNA miR19b, induced metabolic alterations that are similar to the diet-induced phenotype. Furthermore, this pathological phenotype was inherited by the offspring after crosses with healthy partners. Our results indicate that acquired food-induced trait inheritance might be enacted by RNA signalling.

A fundamental law of genetics states that progenies do not inherit adaptive, pathological or neural features acquired in response to environmental conditions. However, recent studies seem to contradict this dogma. Paternal inheritance of diet-induced obesity, diabetes and its associated metabolic disorders, which is a worldwide epidemic and an acute societal problem^1^, was first suggested by epidemiological analysis of human cohorts and later confirmed by experimental analysis. For instance, the offspring of fathers who had been undernourished during the 1944–1945 famine in The Netherlands developed increased adiposity more frequently than controls^2^,^3^ and this up until the second generation. Furthermore in the Overkalix cohort study^4^, a northern Swedish community that endured year-to-year food supply variations had increased diabetes frequencies, which have been related to the grandfathers’ food availability. Moreover, hereditary transmission of diet-induced characteristics was recently confirmed and later analysed by experimental approaches. Female rats born to fathers on a high-fat diet had impaired insulin secretion and glucose tolerance^5^. Another study showed that after maternal exposure to a high fat diet, mice had increased body size and reduced insulin sensitivity and these traits were transmitted up to the third generation^6^,^7^. Furthermore, a study reported that the fat mass of mice raised on a high fat “Western-like” diet steadily increased over four generations^8^. In addition, male and female born to fathers fed a low protein and high sugar diet had a modified liver transcriptome^9^. To finish, C57BL/6 males fed a lipid-rich diet exhibited obesity in the absence of overt diabetes and transmitted the altered metabolic health to their progeny^10^.

The frequent occurrence and heritability of metabolic disorders preclude Mendelian transmission of mutational events and are reminiscent of epigenetic heredity documented in various organisms, ranging from *Caenorhabditis elegans* to humans reviewed in ref.^11^. Interestingly, histone modification and DNA methylation patterns were reported to be altered in the testis and sperm of the affected males^5^,^12^,^13^ and a chromatin-depend signature of paternal-diet-induced intergenerational metabolic reprogramming has been identified^14^. However, the possible roles of these epigenetic marks in transgenerational signaling are still undetermined^15^. Experimental evidence has pointed to small non-coding RNAs (sncRNAs) as a possible vector of epigenetic inheritance. In *C. elegans*, small non-coding RNAs have been shown to induce heritable modifications of several morphological, physiological and behavioral phenotypes^16^,^17^,^18^. In Drosophila, piRNA can trigger multigenerational germline gene silencing^19^,^20^. In mice, we reported a role of sncRNAs in three instances of paternal hereditary epigenetic variations^21^,^22^,^23^. Furthermore, a recent report extended the concept of RNA-mediated paternal heredity to that of the traumatic stress behavioral responses^24^. Based on these data, we hypothesised that paternal inheritance of diet-induced traits could be RNA-mediated. Regarding our previous studies, the main analytical tool was RNA microinjection into one-cell embryos, which is an efficient and well-tolerated procedure. We report here the experimental induction of diet-induced pathologies by injecting either testis or sperm RNA prepared from males raised on a western-like diet into naive one-cell embryos. Identical metabolic disorders were also induced and inherited after micro-injection of a unique microRNA, i.e., mir-19b, that was found to be up-regulated in the sperm of males that were fed a Western-like Diet.

## Results

### Paternal inheritance of obesity and metabolic pathologies induced by a Western-like diet (WD)

**Figure 1:**
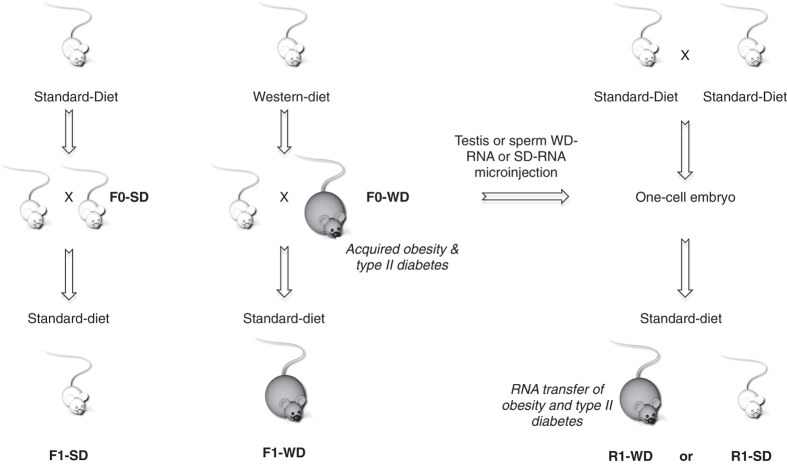
Experimental procedure. (Left part of the panel) 3-week-old C57Bl/6 male mice (F0 mice, Charles River) were divided into two groups. One group (F0-SD) was kept on a standard chow diet (SD) and the other one (F0-WD) was fed a western-like diet (WD) containing 17% casein, 0.3% DL-methionine, 34% sucrose, 14.5% cornstarch, 0.2% cholesterol, 5% cellulose, 7% CM 205B, 1% vit200 and 21% butter. At 16 weeks of age, they were crossed with females that were fed standard diet. The resulting F1 progeny were also fed standard diet. (Right part of the panel) Testis and sperm RNAs, extracted from males raised on either a standard (F0-SD) or western-like diet (F0-WD), were injected into C57BL/6 fertilised eggs collected after normal ovulation, as previously described^22^. After reimplantation in foster mothers, half of the treated eggs developed into normal embryos, designated as the R1 generation. All mice were raised on a standard diet except for the F0-WD males.

**Figure 2:**
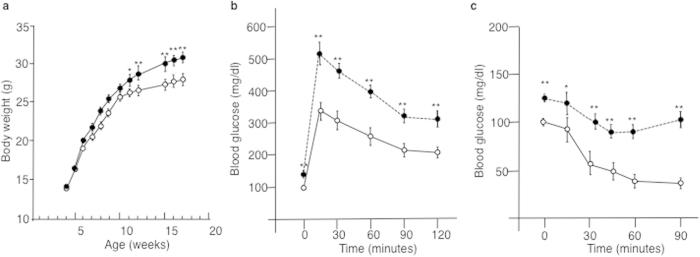
Multiple metabolic alterations in F0 males exposed to western-like diet. (**a**) Body weight of male mice on a control or high-fat diet was measured at 4 months (means ± sem, n = 10 for each group). Males fed a western-fat diet were significantly heavier than the controls. (**b**) Blood glucose tolerance test for 16-week-old males (n = 9 and 10, respectively). Mice were fasted for 12 h before beginning the experiment. Baseline glucose measurements were analysed from tail blood before intra-peritoneal glucose injection (2 mg/g body weight) using the OneTouch Vita system (LifeScan, Johnson & Johnson). Blood glucose measurements were taken from tail blood at 0 min, 15 min, 30 min, 60 min, 90 min and 120 min after injection. (**c**) Blood glucose levels during insulin tolerance test on males at 16 weeks (1 U/kg, n = 9 and 10, respectively. Mice were fasted for 12 h before intra-peritoneal insulin injection (NovoRapid, Novo Nordisk) diluted to 0.08 mU/μl in sterile saline for a final delivery concentration of 0.8 mU/g body weight. Glucose measurements were analyzed as above from tail blood at 0 min, 15 min, 30 min, 45 min and 90 min after injection. White and black circles correspond to mice raised on SD and WD, respectively.

To determine whether RNA molecules can act as epigenetic signals in the hereditary transfer of the disease, a series of experiments was performed according to the general scheme outlined in Fig. 1. In one representative experiment, after weaning, seven C57Bl/6 males were maintained either on a standard diet (F0-SD) or on a high sugar (34% sucrose) and high fat (21% butter) Western-like Diet (F0-WD) for four months (Supplementary Table S1). At 4-month-old, F0-WD animals had body weights greater than F0-SD mice (30.6 ± 1.7 vs. 27 ± 0.9, respectively; p < 0.0001, n = 15) (Fig. 2a). Furthermore, excessive weight gain was associated with insulin resistance, one main feature of type II diabetes. This phenomenon was evidenced by: i) impaired fasting blood glucose levels (at time = 0, F0-WD = 121 ± 25 vs. F0-SD = 93 ± 21 mg/dl; p < 0.001) (Fig. 2b,c), ii) impaired glucose tolerance (evidenced by higher glucose peaks in F0-WD males compared to F0-SD animals; 513 ± 83 vs. 347 ± 69 mg/dl after a glucose tolerance test (GTT), respectively; p < 0.0001, n = 15) (Fig. 2b) and iii) insulin resistance (the decrease in blood glucose levels after insulin injection was less important in F0-WD compared to F0-SD animals after an insulin tolerance test (ITT)) (Fig. 2c).

**Figure 3:**
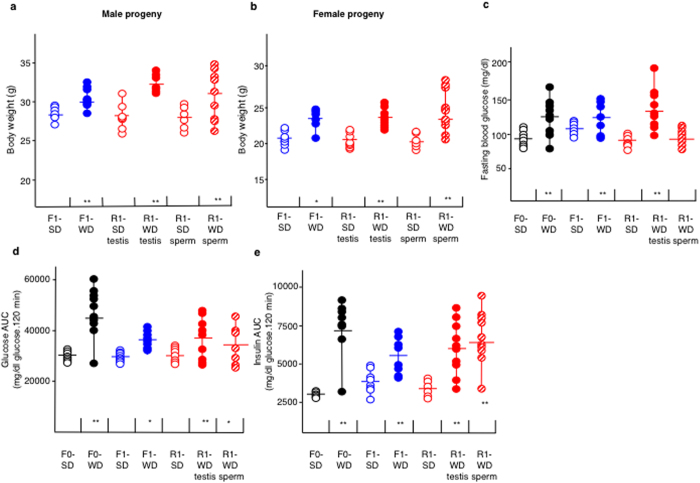
RNA-mediated inheritance of metabolic features of males raised on Western-like diet. Mice were raised on a standard diet except the F0-WD males. Body weights of male (**a**) and female (**b**) derived from either F0-WD or F0-SD fathers or from micro-injected one-cell embryos. (**c**) Fasting blood glucose levels measured as described in Materials and Methods. Areas under the curve (AUC) of the intra-peritoneal glucose tolerance test (AUC-GTT) and of the insulin tolerance test (AUC-ITT) were calculated using the trapezoidal rule^25^ in order to be able to distinguish the response to either glucose (**d**) or insulin (**e**) injections of each mouse individually. All values were recorded at four months of age. F0-SD mice (n = 10); open black circles), F0-WD mice (n = 10; filled black circles). F1-SD (n = 6; open blue circles) and F1-WD (n = 10; filled blue circles) progenies correspond to mice obtained from natural crosses between normal females and males raised on either S or W Diet, respectively. R1-SD (n = 10; open red circles), R1-WD-testis (n = 12; filled red circles) and R1-WD-sperm (n = 10; shaded red circles) correspond to mice derived from fertilised embryos microinjected with SD-RNA testis, WD-RNA testis or WD-RNA sperm, respectively. **p < 0.01.

Then, the F0-WD and control F0-SD males were mated at the age of four months with 8-week-old healthy standard-diet fed females. The males were removed after mating, and both the mothers and progenies were fed a standard diet. As shown in Fig. 3a,b, male and female offspring from WD fathers (F1-WD) exhibited increased body weights (29.7g ± 0.9 and 24g ± 1.5, respectively, n = 13) compared to those from SD fathers (F1-SD) (28.0g ± 0.9 vs. 21.7g ± 1.5, respectively; p < 0.005, n = 14). Furthermore, the F1-WD male progeny exhibited altered fasting blood glucose (Fig. 3c) and higher glycemia in response to both glucose (Fig. 3d) and insulin (Fig. 3e) injections (p < 0.0001 and p < 0.05, respectively; n = 11), determined by calculating the area under the curve (AUC) following an intra-peritoneal glucose tolerance (GTT) and an insulin tolerance test (ITT)^25^.

### Transfer of obesity and metabolic pathologies by microinjection of F0-WD testis and sperm RNAs into one-cell embryos

Based on our previous data^21^,^22^,^23^ and on the fact that the testis is mainly composed of germ cells, we assumed that RNA molecules acting in hereditary transmission must be present in sperm and also in testis. To determine whether RNA could be the vector of paternal epigenetic inheritance of newly acquired metabolic features, we performed microinjection of either sperm or testis RNA from two F0-WD and two F0-SD fathers into one-cell embryos (Fig. 1), in accordance with published procedures^22^,^25^. Forty-six pups were born from embryos injected with either testis or sperm RNAs from F0-WD males, designated as R1-WD-testis (14 males, 13 females) and R1-WD-sperm (9 males, 10 females) and 26 pups were born from embryos injected with either testis or sperm RNA from F0-SD males, designated as R1-SD-testis (7 males, 7 females) and R1-SD-sperm (6 males, 6 females). Strikingly, the body weight of the R1-WD-testis and R1-WD-sperm males was significantly greater compared to their R1-SD-testis and R1-SD-sperm respective controls (32.38 g ± 1.3 vs. 28.03 g ± 1.3 and 31.22 g ± 4.79 vs. 27.8 g ± 3.2, respectively, p < 0.01; n ≥ 9; Fig. 3a). The female R1-WD-testis and R1-WD-sperm were also heavier than their R1-SD-testis and R1-SD-sperm counterparts (24 g ± 1.3 vs. 21.5 g ± 0.9 and 23.3 g ± 3 vs. 21.1 g ± 2.3, respectively, p < 0.01; n ≥ 6; Fig. 3b) To complement the above experiment, we also tested whether R1-WD-testis and R1-WD-sperm progenies exhibited signs of insulin resistance. Interestingly, we found that the R1-WD-sperm and R1-WD-testis animals exhibited altered glucose tolerance and impaired insulin sensibility as analysed by area under the curve analysis (p < 00.1, n = 7; Fig. 3d,e, respectively). We also found that R1-WD-testis males exhibited fasting blood glucose levels that were significantly greater than the R1-SD males (120.4 mg/dl ± 35.5 vs. 85.44 mg/dl ± 22.36; p < 0.05, n ≥ 7), which was not the case with the R1-WD-sperm males (85.44 mg/dl ± 22.36 vs. 95 mg/dl ± 16.88; p > 0.1, n ≥ 7) (Fig. 3c).

### Transcriptome alterations in Western-like diet testis and sperm cells

Of two comparable RNA populations isolated from the same tissue, one efficiently induced an epigenetic change (RNA from WD mice), whereas the other RNA population did not (RNA from SD mice). We inferred that available sequence analysis technologies would pinpoint critical differences between the two RNA preparations. First, we performed a comparison of the WD and SD testis mRNA expression profiles by oligonucleotide microarray hybridisation. The analysis was performed on three randomly chosen animals of each group, however those who exhibited the strongest effects of diet were excluded from the selection group. Differentially expressed transcripts, either up- or downregulated, were detected in the WD males through Volcano and MA plot representations (Supplementary Fig. S1). The resulting list (Supplementary Table S2) provided only limited information, as changes were often subtle in magnitude (~50%), and no obvious candidate for a master gene function was identified.

As small noncoding RNAs of mice and non-mammalian model organisms were found in several instances to act as hereditary epigenetic determinants (i.e., small non-coding RNAs (sncRNA), microRNAs (miRNA) and piwi-interacting RNAs (piRNA)), reviewed in^11^, we extended the transcriptome analysis of the testis RNAs to the small (less than 200 bp) fraction. Deep sequencing analysis revealed the differential expression of 13 miRNA (p ≤ 0.1; fold change ≤ ±1.5; Table 1) and 190 piRNAs (p_value_ ≤ 0. 05; p_adjustvalue_ ≤ 0.05; fold change ≤ ±1.5, Supplementary Tables S3) which are spread across 63 different piRNA clusters (Supplementary Tables S4). In the resulting list of differentially expressed small RNAs, we checked whether some of them had been previously implicated in obesity and metabolic pathologies. To the best of our knowledge, no piRNA has been previously implicated in these metabolic disorders, whereas several microRNAs had been previously associated with obesity and/or diabetes or were reported to be overexpressed in comparable unhealthy-diet animals^10^,^26^,^27^. Therefore, we focused our study on microRNA analysis. By quantitative RT-PCR analysis, we confirmed the deregulation of miR-182, miR-19a, miR-19b, miR-29a and miR-340 in testis and sperm RNA of the WD males compared to SD males (Table 1). The observed fold-change difference in miR-183 and let7i content between testis and sperm may be due to the heterogeneous cell composition of testis. Indeed, although testis is mainly composed of germ cells, the contribution of somatic cells to this deregulation might be more or less important depending on the miRNA analysed.

### Induction of obesity by microinjection of the microRNA miR-19b into one-cell embryos

**Figure 4:**
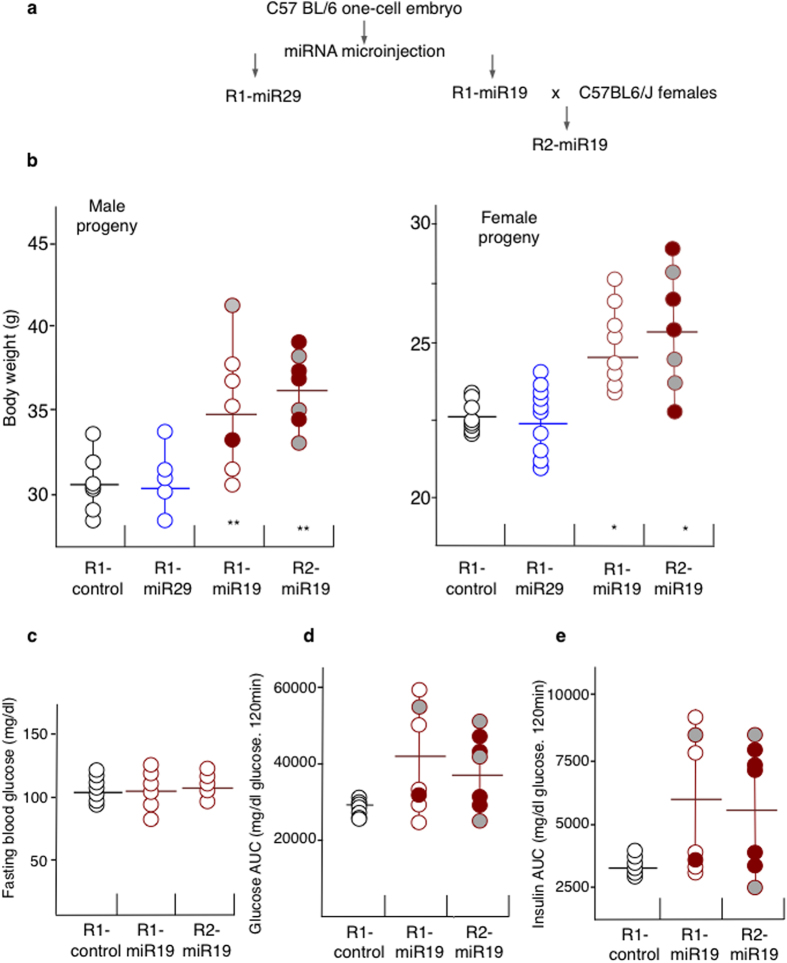
Injections of miR-19b into one-cell embryos induced obesity and aspects of the diabetic phenotype. (**a**) miR-19b and miR-29a were microinjected into one-cell embryos and the derived mice were named R1-miR19 or R1-miR29, respectively. At 16 weeks of age, two R1-miR19 male were crossed with females to obtain the R2-miR19 progeny. All mice were fed a Standard diet. (**b**) Body weights of 4-month-old males and females derived from the microinjection of no-RNA (R1-control; black circles), miR-29 (R1-miR29, n = 15; blue circles) or miR-19b microRNA (R1-miR19 (n = 7) and R2-miR19 (n = 7); red circles) into one-cell embryos. All mice were raised on the standard diet. (**c**) Fasting blood glucose levels. (**d**) Area under the curve of blood glucose after a glucose tolerance test. (**e**) The area under the curve of blood glucose during an insulin tolerance test on males at 16 weeks is shown. The areas under the curve (AUC-GTT) during the glucose tolerance test and during the insulin tolerance test (AUC-ITT) were calculated using the trapezoidal rule. The R1-miR19 fathers and their respective R2-miR19 progenies are indicated by grey-filled or red-filled circles.

Our differential RNA-Seq analysis identified miR-19b and miR-29a as the two most abundant deregulated microRNAs in WD mice (Table 1). These two microRNA candidates were, therefore, injected separately into fertilised one-cell embryos to test for their potential as inducers of obesity and metabolic disorders (Fig. 4a). Strikingly, males and females born from miR19b-microinjected one-cell embryos (designated R1-miR19) had, in average, body weights greater than the controls (34.17g ± 6.5 vs. 31g ± 0.5 and 26.49g ± 3 vs. 22.81g ± 0.76, male and female progenies, respectively), and these weights were similar or higher levels than the ones of animals raised on western-like diet and their F1 and R1 progenies (Fig. 4b). By contrast, the body weights’ distibution of 15 individuals that had received miR-29a were identical to mock-injected controls, at 4-months. None of the R1-miR19 mice, however, showed increased values of fasting glucose levels (Fig. 4c), which is at odds with the more complete pathology observed for the WD series, but is similar to the R1-sperm injected progenies and to the previously published reports of metabolic alterations without overt diabetes^10^.

The degree of glucose intolerance and insulin resistance was more variable, as only half of the obese R1-miR19b animals showed impaired glucose tolerance and insulin sensitivity (Fig. 4d,e). The normal or subnormal values exhibited by the other half correspond to the variable penetrance of an abnormal phenotype^28^. This phenomenon was made apparent by analysis of the progenies of individual R1-miR19 males crossed with control females, namely the R2-miR19 progenies (Fig. 4e). Indeed, it is interesting to note that some R2-miR19 mice developed the full miR19 phenotypes despite the normal metabolic features of their R1-miR19 progenitors.

## Discussion

Identifying the nature of the vector involved in the inheritance of obesity and type II diabetes is essential to understand the progression of these major biological and medical problems. Our work provided the first evidence that perturbation of the zygote’s RNA content by microinjection of specific RNA induced zygote’s reprogramming which led to the development of the pathological traits in the adult. Given that the effect was specifically observed when the incoming RNA was prepared from the affected individual’s germ cells, it can be reasonably inferred that the transmission of the disease is initiated by sperm RNA transfer at the time of fertilisation. One caveat of our experiments is that microinjection differs from fertilization in many respects, such as the higher number of injected molecules compared to that present in sperm. This difference may be compounded by the observation in comparable experiments that a high amount of injected RNA is excluded from the nucleus and eliminated within the first minutes, after injection^21^.

The clear difference between the R1-WD and R1-SD animals indicates that RNA carried by the sperm into the oocyte is able to modify gene expression in the resulting animals. However, although F1-WD and R1-WD mice were clearly different from the F1-SD and R1-SD controls, they showed a significant degree of individual variation compared with the more uniform F0-WD parent phenotypes. This individual variation was also evident in R1-miR19 mice. Indeed, some R1-miR19 displayed a relatively low body weight and a normal glucose regulation, whereas their respective R2-miR19 progeny displayed the full disorder (increased body weight, altered blood glucose levels and insulin tolerance). Theses results suggest that upon RNA injection, epigenetic changes take place which can remain phenotypically silent but are later transmitted to the progeny (R2-miR19 obese males were born from R1-miR19 normal males) (Fig. 4). In a previous study of diet-acquired metabolic abnormalities^9^, similar variations were qualified as ‘variable penetrance’ which may be related to the interindividual variability of gene expression first described as a ‘rheostat effect’ for imprinted genes^28^. The rheostat effect appears to be a frequent feature of epigenetic alterations; furthermore, it is interesting to note its presence both in RNA-mediated induction of the disease and in the sexual F1-WD progenies, suggesting a common mechanism.

We have established that RNA is a vector of epigenetic information; however, the mechanism that is initiated in the early embryo and leads to the establishment of a complex inheritable pathology remains to be determined. Only partial indications can be gathered from previous studies. In three instances^21^,^22^,^23^, the expression level of a key regulatory gene was modified at the transcriptional level and was variable among individuals (inter-individual variability) and at defined developmental stages. RNA specification is not contradictory to other reported modes of expression control, namely chromatin structure and/or DNA methylation patterns, whose variations are the most likely modes for gene expression adjustments. To explain the strict locus-specificity of the effect, sequence recognition appears as a likely, if not unique mechanism, in which short ncRNAs can play critical roles. An epigenetic mark has to be established on one or several key regulators at the one-cell stage embryo, eventually leading to structural changes and metabolic alterations at later times. Identification of targets, critical to understanding the whole process, is challenging. We have identified miR-19b as one of these molecules, but further assessment of its specific role is complicated by the pleiotropic nature of its activity, spanning from lymphomagenesis^29^ to the inflammatory response^30^. Further investigation on the miR-19 mice through the transcriptomic analysis of either early embryo and/or adipose-derived stem cells will provide additional insight into the mechanisms of miR-19 action and will help to identify genes involved in the development of obesity and/or metabolic disorders at the very early stages of development.

However, considering the high number of molecules micro-injected (about 4000 as previously described in^21^), which probably does not truly and completely mirror the level of microRNA present in WD sperm, it seems unlikely that individual RNAs are responsible for the whole phenotype. As such, we cannot completely exclude the possibility that other small RNAs may play a role in the inheritance of diet-induced metabolic phenotypes. This could more particularly apply to piRNAs, whose expression is for some of them altered in WD-sperm. All the more so as this RNA population has been demonstrated to play a role in the paramutation phenomenon (an epigenetic interaction between two alleles of a locus) in non-vertebrate animals^31^ and to be deregulated at specific piRNA clusters in another example of RNA-mediated epigenetic inheritance^24^.

There is a growing number of examples of RNA-mediated epigenetic heredity, not only in invertebrate models reviewed in^11^, but also in mice in whom the acquired features cannot be accounted for in Mendelian terms, such as the diet-induced syndrome and the heritable behavioural response to early traumatic stress^24^. These results reinforce the idea that RNA-mediated controls of epigenetic regulations may play determinant roles in embryonic development.

## Methods

### Mice

The experiments described were performed out in compliance with the relevant institutional and French animal welfare laws, guidelines and policies. The experiments were approved by the French ethic committee (‘Comité Institutionnel d’Ethique Pour l’Animal de Laboratoire’; file number NCE/2012-54). Mice were maintained with the C57BL/6 genetic background (Charles River, Wilmington, Ma, USA). Male 3-week-old mice were divided into two groups. One group was kept on standard chow and the other was fed a western diet containing 17% casein, 0.3% DL-methionine, 34% sucrose, 14.5% cornstarch, 0.2% cholesterol, 5% cellulose, 7% CM 205B, 1% vit200 and 21% butter (Diet Western 1635, Safe, Augy; France; Supplementary Table S1).

### Glucose tolerance test

Mice were fasted for 12 h before the experiment. A solution of sterile 0.2 g/ml glucose was freshly prepared in 0.9% sterile saline. Baseline glucose measurements were analysed from tail blood before intraperitoneal (ip) glucose injection (2 mg/g body weight) using the OneTouch Vita (LifeScan, Johnson & Johnson, Milpitas, CA, US) system. Blood glucose measurements were taken using tail blood at 0 min, 15 min, 30 min, 60 min, 90 min and 120 min after injection. Areas under the curve during the insulin tolerance test (AUC-ITT) were calculated using the trapezoidal rule.

### Insulin tolerance test

Mice were fasted for 12 h before the experiment. Baseline glucose measurements were analysed using the OneTouch Vita (LifeScan, Johnson & Johnson company) system from tail blood before ip insulin injection (NovoRapid®, Novo Nordisk, Bagsværd, Denmark) diluted to 0.08 mU/μl in sterile saline for a final delivery of 0.8 mU/g body weight. Blood glucose measurements were taken using tail blood at 0 min, 15 min, 30 min, 45 min, 60 min and 90 min after injection. Areas under the curve during the glucose tolerance test (AUC-GTT) were calculated using the trapezoidal rule^25^.

### RNA preparation and microinjection

Frozen tissue was placed in 10 volumes of ‘RNAlater-ICE’ (Applied Biosystems, Life Technologies, Carlsbad, CA, USA) on dry ice^32^. The samples were stored overnight at −20 °C. RNA was then extracted either by the TRIzol procedure (Invitrogen, Life Technologies) or using the RNA easy Mini Kit (Qiagen, Hilden, Germany). The same preparations of testis RNAs were used for microinjection, sequencing and microarray analysis. The quality of the RNA preparations was verified by spectrometry on the Agilent Bioanalyzer 2100 apparatus (Agilent, Santa Clara, Ca, USA; Supplementary Fig. S2). Microinjection into fertilised eggs^25^ was performed according to established transgenesis methods^22^. RNA solutions were adjusted to a concentration of 1 μg/ml and 1–2 pl were microinjected into the pronucleus of C57BL/6 fertilised eggs isolated after normal ovulation and mating. Synthetic microRNA, miR-19b (CUGUGCAAAUCCAUGCAAAACUGAC) and mir-29a (GACUGAUUUCUUUUGGUGUUCAGA)(purchased from Sigma, Saint-Louis, MO, USA) was prepared in filtered microinjection buffer (10 mM Tris, pH 7.4; 0.1 mM EDTA) at a concentration of 4,000,000 molecules/pl.

### RT-PCR and quantitative RT-PCR

miRNA quantitation was performed using miScript Primer Assays (Qiagen), following the manufacturer-recommended protocols. Real-time PCR was performed with a Light Cycler Instrument (Roche Diagnostics, Indianapolis, IN 46256, USA) using the miScript SYBR Green PCR kit (Qiagen).

### Microarray analysis and sequencing

Microarray analysis of the expression of 45281 transcripts was performed by the Microarray Service CS3021 Illumina Gene Exp Process (MouseWG-6 v2.0 Expression BeadChips, Qiagen), and small RNA high-throughput sequencing and analyses were performed by the Functional Genomics Platform, Institut de Pharmacologie Moléculaire du CNRS, Sophia Antipolis, France. The supplied samples have been sequenced on SOLiD 5500XLHigh Throughput Sequencer. The obtained libraries of sequences (reads) were aligned with the mouse genome (mm9) using Bowtie aligner with the following parameters: Reads trimming at the adaptator position (trimming at 31 bases otherwise) and 2 allowed mismatches over the total read length. The following public databases were used for the read annotation process : miRBase miRNAs data base mirbase18, Rfam database and ncRNA.orgdatabase. The normalised, subtracted background and expression (Robust Microarray Analysis, RMA) data were further analysed using Bayesian model moderated *t*-test implemented in the Bioconductor *limma* package of the statistical software R (http://www.r-project.org/)^33^. The false discovery rate [FDR, (FDR-correction p < 0.05)] was used to adjust p-values and to correct for the multiple testing issues^34^. After conversion of the deregulated piRNAs with genomic coordinates from mm9 to mm10 using web-based LiftOver tool located at http://usegalaxy.org^35^, deregulated piRNAs were mapped to known clusters using the piRNA cluster data base provided on the web site http://www.smallrnagroup.uni-mainz.de^36^,^37^.

### Statistical analysis

Because of the rheostat-type (non-normal) distributions and the limited number of animals^28^, non-parametric methods were used, and a median value comparison was performed by Wilcoxon rank analysis^38^. Statistical analyses were with an unpaired t test with Welch’s corrections. Data are expressed as the median with p values <0.05 considered to be statistically significant.

## Additional Information

**How to cite this article**: Grandjean, V. *et al.* RNA-mediated paternal heredity of diet-induced obesity and metabolic disorders. *Sci. Rep.* **5**, 18193; doi: 10.1038/srep18193 (2015).

**Data availability**. All microarray and deep sequencing data used in this study have been deposited in GEO (http://www.ncbi.nlm.nih.gov/geo/) under accession numbers GSE44301 and GSE43555.

## Supplementary Material

Supplementary Information

## Figures and Tables

**Table 1:** Differentially expressed microRNAs in testis and sperm of Western vs. standard diet fed males.

Name	Read number		Fold change		Reference
	RNA-seq testis	RNA-seq testis	q-RT miRNA testis	q-RT miRNA sperm	
Downregulated miRNAs					
mmu-miR-183-5p	24	1.95*	1,8^†^	1,3	10,39
mmu-miR-182-5p	54	1.75**	2^†^	1,7^†^	39
Upregulated miRNAs					
mmu-miR-124-3p	103	1.95*	ND	ND	40,41
mmu-miR-30e-5p	421	1.95*	1,1	1,2	40
mmu-miR-468-5p	78	1.65*	ND	ND	40,41
mmu-miR-29a-5p^§^	8161	1.5*	1,5^†^	1,3^†^	40,41
let-7i-3p^§^	41	1.6**	2	1	40
mmu-miR-340-5p	61	1.5**	1,5^†^	1,7^†^	26,42
mmu-miR-146a-5p	21	1.8*	ND	ND	41
mmu-miR-376b-5p	15	1.8*	ND	ND	40,41
mmu-miR-19b-3p^§^	16211	1.5*	1,8^†^	1,9^†^	40,41
mmu-miR-19a-3p^§^	3058	1.5*	1,8^†^	1,9^†^	40,41
mmu-miR-101b-3p	344	1.5**	ND	ND	27,41

Validation of selected differentially expressed miRNA (*p < 0.05, **p < 0.01) by quantitative reverse transcription polymerase reaction. Values are miRNA expression levels relative to internal controls (n = 10 for each group). SNORD61 was used for normalisation because of its stable expression in Western Diet vs. Standard Diet. The p value was calculated using the t test between western and standard diet samples ^†^p < 0.05). The column “Reference” indicates the published articles showing an association between the indicated miRNA and the development of obesity and/or diabetes in different systems.
