# Supplementary material for: RNA-mediated paternal heredity of diet-induced obesity and metabolic disorders
Source: Sci Rep. 2015 Dec 14;5:18193. doi: 10.1038/srep18193 (PMC4677355; doi:10.1038/srep18193)
Supplement: Supplementary Information [file srep18193-s1.pdf]

RNA-mediated paternal heredity of diet-induced obesity and metabolic disorders

Valérie Grandjean[1,2,3 $], Sandra Fourré[4], Diana Andrea Fernandes De Abreu[5], Marie-Alix Derieppe[1,2,3], Jean-Jacques Remy[5] and Minoo Rassoulzadegan[1,2,3]

[1] Inserm, U1091, Nice, F-06108; [2] CNRS, UMR7277, F-06108, France; [3] University of Nice-Sophia Antipolis, UFR Sciences, Nice, F-06108; [4] Institut de Pharmacologie Moléculaire et Cellulaire (IPMC), UMR 6079 CNRS-UNSA, Sophia Antipolis, France; [5] UMR Inra 1355, CNRS 7254, UNS, Sophia Antipolis, France.

[$] Corresponding author:
University of Nice Sophia Antipolis (UNS)
Faculty of Sciences, Parc Valrose,
Institut de Biologie Valrose, iBV
UMR CNRS 7277/Inserm 1091/University Nice Sophia Antipolis
BP 2135
06108 NICE CEDEX 2, FRANCE

Phone : +33 4 92 07 64 09
Fax : +33 4 92 07 64 02

Email address: grandjea@unice.fr

Supplementary Information

- Supplementary Figure Legends
- 2 supplementary Figures (S1 through S2)
- 4 supplementary Tables (S1 through S4)
- Supplementary references

Supplementary figure legends

Supplementary Figure S1. **Volcano Plot.** The Log Odds (or B value) on the x-axis is the odds (or probability) that the gene is differentially expressed between Standard and Western-like Diet testis. A log Odds value of 0 corresponds to 50-50 chance that the gene is differentially expressed. The y-axis indicates the log2 value of fold changes between the two conditions. B. MA Plot. The x-axis is the average expression level among the two conditions, indicating the basal expression level. And the y-axis is the fold change (log2 scale), which indicates the difference level between the two. Each gene is represented on the plot as a single dot. Genes, up- or down-regulated in the Western-like Diet compared to the Standard Diet, correspond to blue spots and are listed in Table S2.

Supplementary Figure S2. **Spectrometric RNA quality checks.** RNAs prepared for microinjection were verified using Agilent Bioanalyser 2100 apparatus. The profiles indicate that both standard and western sperm RNA samples contain no apparent ribosomal RNA which are consistent with pure RNA sperm preparation.

Supplementary references

1 Roovers, E. F. *et al*. Piwi proteins and piRNAs in mammalian oocytes and early embryos. *Cell Rep* **10**, 2069-2082 (2015).

2 Rosenkranz, D. & Zischler, H. proTRAC--a software for probabilistic piRNA cluster detection, visualization and analysis. *BMC Bioinformatics* **13**, 5 (2012).

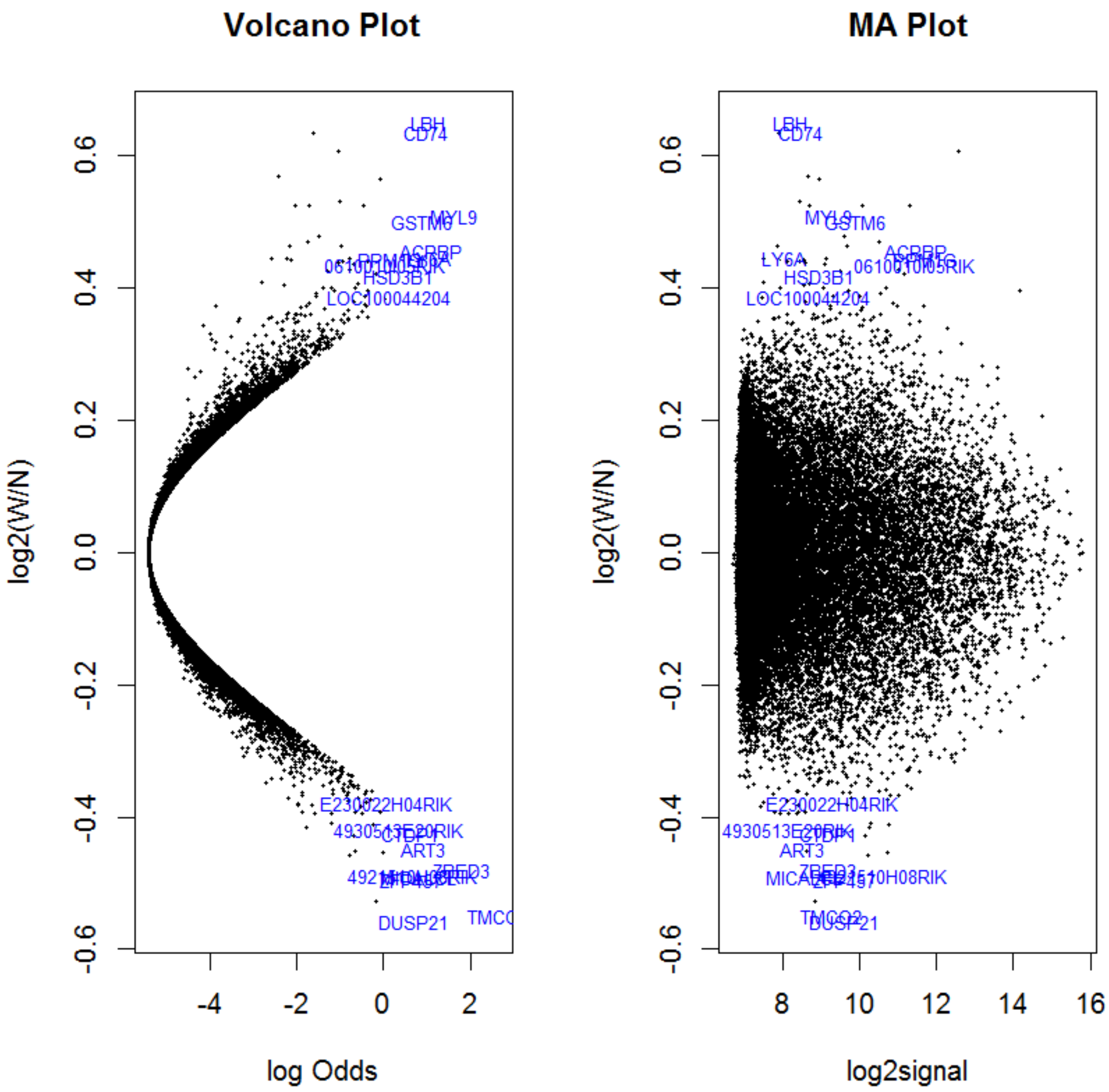

Supplementary Figure S1. Grandjean et al.

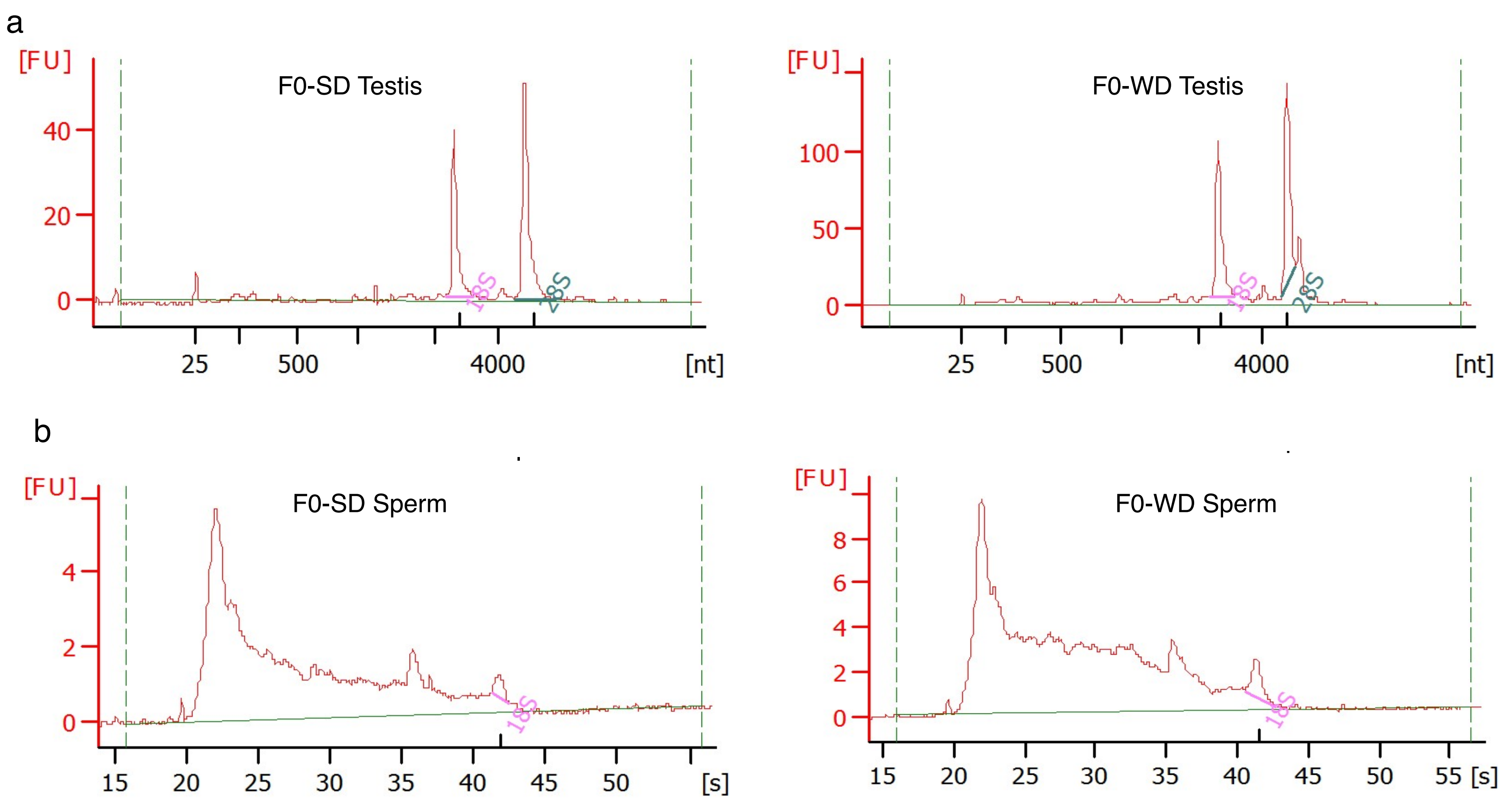

Supplementary Figure S2 Grandjean et al.

Table S1 Experimental diet nutritional composition

	Standard Diet[a]	Western Diet[a]
Carbohydrates (kcal%)	59,9 (of which sugar 3,2%)	46,1 (of which sugar 34%)
Proteins (kcal%)	16,1	15,3
Lipids (kcal%)	3,1	17,9
Caloric intake (kcal/kg)	2791	4035

[a]Standard Diet and Western Diet formulation according to SAFE

Supplementary Table S2. **Genes up- or down-regulated in the Western Diet compared to the Standard Diet.**

TargetID	IlluminaProbe	log2signal	log2(W/N)	p-value	N	N3	N4	W	W2	W4
**DUSP21**	**6480196**	9,58580985	-0,5573977	8,37E-05	712	1085,7	1242,9	658	540,8	621,6
**TMCO2**	**1170377**	9,25456911	-0,550455	3,91E-06	655,4	840,3	861,2	484,6	488,6	471,9
**OTTMUSG000**	**5130528**	8,81610352	-0,5273555	0,00029025	524	688,3	510,6	420,7	285,6	384,8
**ZFP457**	**6420563**	9,62767069	-0,4944659	8,59E-05	877,5	1199,4	931,9	682,4	573,3	653,8
**MICALCL**	**6520224**	8,5827955	-0,4909786	6,86E-05	361,7	635,5	473,7	310,4	344,5	277,2
**4921510H08R**	**4900639**	10,5933185	-0,4878514	8,20E-05	1527,8	2663,4	1809,3	1242,1	1265,8	1178,4
**ZBED3**	**1740161**	9,20381296	-0,4792123	1,50E-05	683,5	724,2	793,4	474,7	488,1	467,7
**PSMD6**	**6180088**	10,2093418	-0,4584601	0,00072592	1081,1	1836,1	1619,9	979,6	1210,3	727,8
**1700015E13R**	**3940408**	10,722115	-0,4538694	0,0002383	1392,4	2593,9	2594,5	1247,9	1347	1485,1
**LOC213411**	**4670632**	8,60459717	-0,4523619	0,00059628	410,8	671,2	391,3	365,9	319,2	276,3
**ART3**	**7000500**	8,50666247	-0,4484799	5,82E-05	336,5	553,4	477,2	257	380,1	271,9
**HIPK4**	**2350546**	10,1310882	-0,427455	0,00062764	862,2	1829	1668,2	818	1018,2	910,5
**CTDP1**	**520746**	9,18156581	-0,4253574	9,21E-05	632,9	765,1	732,6	499,5	459,6	478,8
**4930513E20R**	**460187**	8,15378688	-0,4183476	0,00013285	281,1	394,2	364,3	222,5	234,1	254,7
**SUGT1**	**580152**	10,7660794	-0,4114791	0,00033175	1563	2793	2226,8	1361,5	1503,1	1402,8
**NMT2**	**6450373**	7,93415203	-0,3940891	0,00047263	226	384,4	285,8	182,3	217,7	219,4
**MED7**	**6420156**	7,76468368	-0,3931397	0,00059852	228,2	305,8	246,9	172	173,3	206,5
**TMEM209**	**6420367**	8,58318223	-0,3916577	0,00025476	350,9	551,1	506,8	290,8	355,8	319,8
**A430037M23I**	**6060544**	7,43603018	-0,3833058	0,00049556	167,2	216,7	239,6	135,2	164,9	142,5
**E230022H04R**	**4880390**	9,31165764	-0,379095	0,00020248	762	798,1	736,7	562,3	528,1	504,5
**LOC10004783**	**6130202**	7,50182954	-0,3769548	0,00041841	195	226,8	219,9	141,4	183,4	141,2
**UCHL5IP**	**4570079**	8,11939124	-0,3759743	0,00074363	257,9	411,7	338,2	230,8	252,2	221,8
**RHBDL2**	**2970307**	10,7146008	-0,3726464	0,00036209	1728,8	2221,6	2189,7	1423,9	1420,5	1349,5
**ATP5SL**	**5270131**	9,77181887	-0,3720594	0,00054409	710,8	1331,1	1234	566,3	758,5	891,9
**SOX5**	**5050082**	11,0081401	-0,3682525	0,00077092	2282,3	2958,8	2302,2	1946,2	1631,3	1547,4
**ZFHX3**	**150605**	7,76360962	-0,3649002	0,00043974	209,6	313,6	255,7	173,4	207,9	176,3
**WDR35**	**5080072**	9,55754216	-0,3620437	0,00074968	750,9	1039,5	946	607,2	598,8	695,1
**KHSRP**	**4920593**	8,39745294	-0,3616897	0,00084951	301,5	463,1	460,3	248,8	273,9	342,8
**LOC10004067**	**2940673**	10,5172777	-0,3612726	0,00041008	1714,1	1770,5	1824,1	1339,9	1079,4	1260,8
**FANCL**	**6620240**	8,37418001	-0,3608961	0,00034809	343,6	436	404,1	261	296,4	287,6
**TIMP3**	**1400309**	8,91589322	0,35602822	0,00054995	369,6	520,3	461,9	422	655,7	506,8
**LAMB2**	**6840209**	7,89516815	0,35652882	0,00038822	163,7	272,6	235,2	195,9	328,4	269,9
**1700029F12R**	**1770504**	11,2915265	0,35995174	0,00086015	2524,9	2248,3	2319,5	2832,7	2221,7	3040,8
**AMHR2**	**3310739**	8,52406641	0,36366088	0,00047346	307,7	411,2	305,2	339,2	470,2	397,5
**CD151**	**1400630**	10,0151647	0,36677298	0,00075086	798,1	1122,2	1005,9	817	1356,5	1219,8
**SPTLC1**	**450048**	9,21445583	0,37225431	0,00041237	447,6	612,9	605,5	588,8	733,2	607,9
**ALDH1A2**	**6330008**	8,86333918	0,37434794	0,00042977	384,4	425,1	478,2	413,6	549,1	564
**MRPL4**	**4810100**	8,56874317	0,37854329	0,00063127	317,7	350,1	378,3	323,6	428,2	511,4
**GLOXD1**	**6480609**	7,46384962	0,38515349	0,00020998	123,3	193,3	171,6	156,9	241,1	195
**LOC10004420**	**3830048**	8,67174533	0,38627035	0,00018334	279	489,5	380,3	323,4	581,1	466,2
**LOC10004650**	**4610397**	9,28995922	0,38718891	0,00042973	480,5	640,1	624,2	549,8	666,3	858,1
**DEFB19**	**2030088**	14,1621756	0,39594323	0,00039391	15501,3	20021,7	15849,5	16406,8	25160	18816,3
**TGM2**	**7000187**	9,06624757	0,40106689	0,00059245	340,1	607,5	576,7	396,6	875,1	565,4
**ATP6V0D1**	**6290673**	8,70114094	0,40676388	0,00053496	346,2	364	425,1	343,9	522	531,1
**HSD3B1**	**360463**	8,94033029	0,41807307	0,00013645	382	489,9	470,3	493,5	648,1	493,5
**SLC25A5**	**5870092**	11,1597012	0,42048999	0,0002995	1814,5	2316,3	2223,7	1865,2	3058,4	2653,4
**EG668668**	**7570762**	11,0204684	0,42660908	0,00069992	1603,9	1901	2268,5	1710,1	2876,5	2314
**0610010I05RI**	**3990427**	11,4512637	0,43483371	0,00020984	2889,8	2495,8	2352,3	2987,6	3011,7	3171,2
**INHA**	**240739**	8,5961062	0,43813215	0,00103142	348,1	306,1	390	348,8	529	430,5
**PPM1G**	**3370546**	11,6981224	0,44411152	0,00018829	3492,3	2588,7	3114,6	3632,6	3346,5	3964,7
**LY6A**	**1690187**	8,02745599	0,44528579	4,91E-05	185,2	297,7	225,1	218,1	405,5	280,8
**ACRBP**	**2320017**	11,4902449	0,45586514	4,42E-05	2786,5	2404,2	2708,5	3214	2993,7	3291,4

The experimental design is shown in Figure 1. Total RNAs from animal testes at four months old were prepared and subjected to analysis (Microarray Service CS3021 Illumina Gene Exp Process Ref: 338908 Qiagen). The resulting microarray data compared three males fed the WD diet (W, W2 and W4) to standards (N, N3 and N4). The RNA levels of WD-fed mice were compared to controls. Colon D ratios (W/S): colour ranged from green for lower expression to red for higher expression in WD.

Supplementary Table S3 **List of differentially expressed piRNA**

FR271924	piRNA	TGAGGAAGCATGAACACG	chr1	57410225	57410252	-1,6239251	0,00022246	0,00671165	207,020497	186,982214	190,928917	182,46599	303,380414	176,107161	173,123457	174,362848
FR233758	piRNA	TGGTACTGGTAACGGGAC	chr1	94890676	94890701	1,61132844	0,00120302	0,01894317	4,40469142	7,10059042	3,74370426	7,53299959	4,62470143	4,31635199	3,39457758	7,4726935
FR203286	piRNA	TGAGCTTATTGCAGGATTG	chr1	93452436	93452464	1,72419863	0,00512951	0,04807624	0,88093828	4,73372695	1,24790142	4,18499977	5,54964172	4,31635199	11,8810215	6,22724458
FR282027	piRNA	TTCTTATGATTATTCAGGG	chr1	94893438	94893465	1,86448223	0,00514074	0,04810544	58,1419267	31,9526569	62,395071	78,6779957	43,4721935	44,8900607	52,6159525	41,0998142
FR298886	piRNA	CTATAGATCTTGTGGACAG	chr10	31317240	31317258	-3,4642917	0,00110737	0,01811778	3,52375313	4,73372695	2,49580284	5,85899968	9,24940286	6,90616319	3,39457758	9,96359133
FR044731	piRNA	TAGATTTTATGTCTATGTTT	chr10	66159724	66159746	-1,6541241	4,90E-11	2,24E-07	61,6656798	86,3905168	87,3530993	65,2859964	136,891162	101,865907	87,4103727	83,4450774
FR344724	piRNA	TAATAGTCTGTGTGACGTG	chr10	66149908	66149935	2,74388502	0,00137649	0,02063078	10,5712594	10,6508856	9,98321135	7,53299959	5,54964172	14,6755968	15,2755991	7,4726935
FR114471	piRNA	TAGCATTACAAATTGTTAA	chr10	62145467	62145496	1,51817421	0,0002788	0,00773078	45,8087907	61,5384503	36,1891412	45,1979975	42,5472532	33,6675455	50,0700193	53,5543034
FR132075	piRNA	TCAGAGATCAGAGAAAAA	chr10	85214749	85214778	1,77849845	0,00307289	0,03437017	9,69032112	3,55029521	1,24790142	7,53299959	0,92494029	4,31635199	11,8810215	1,24544892
FR273679	piRNA	TACCAGAACATATTTGGCT	chr10	62149748	62149778	1,55647618	2,85E-06	0,00048374	5,2856297	4,73372695	2,49580284	8,36999954	1,84988057	6,04289279	5,09186637	6,22724458
FR233265	piRNA	TGATTCTCAGGCTTTGCTG	chr10	85227755	85227783	1,60849674	1,17E-05	0,00106387	20,2615805	35,5029521	27,4538312	30,1319984	11,0992834	26,7613823	14,4269547	18,6817337
FR157995	piRNA	TTTTAAATTTGAGCTGATCT	chr10	66159744	66159771	1,66363957	0,0007271	0,01401614	66,0703713	44,970406	46,1723525	85,3739953	46,2470143	56,1125759	49,2213749	54,7997523
FR250874	piRNA	TTATGTTAAAAGAAAAGTT	chr10	86039371	86039399	1,6669588	3,02E-05	0,00192624	277,495559	450,887492	243,340777	307,178983	167,414192	251,211686	254,593319	212,971765
FR009778	piRNA	TCTTGTTGGCATTAAAGCC	chr10	86035109	86035138	1,70755457	0,00072974	0,01404935	11,4521977	5,91715869	6,2395071	17,576999	3,69976115	10,3592448	7,63779956	16,1908359
FR252743	piRNA	TCATCATTGCTGAATCTTCA	chr10	66160110	66160138	1,831149	0,00010763	0,00409782	9,69032112	4,73372695	9,98321135	6,69599964	5,54964172	5,17962239	11,8810215	13,6999381
FR199497	piRNA	TACAACTTGAAGAGTATTG	chr10	85227017	85227041	1,87548435	0,00073899	0,01411746	31,7137782	28,4023617	21,2143241	33,4799982	15,7239849	25,8981119	39,8862866	27,3998762
FR091729	piRNA	TATGATGAAATTTACATGC	chr10	85314373	85314401	1,92113203	0,00093354	0,01637549	49,3325439	37,8698156	33,6933383	29,2949984	12,949164	24,1715712	36,491709	22,4180805
FR084166	piRNA	TAAATACAGTAAAATTTGT	chr10	127135716	127135745	2,59665787	0,0011795	0,01879916	22,9043954	21,3017713	26,2059298	15,0659992	37,9225517	33,6675455	26,3079763	18,6817337
FR223095	piRNA	TAGGTTGATGCACCTTCAG	chr10	94136855	94136885	3,41035636	0,00125813	0,01946883	77,5225689	75,7396312	76,1219866	62,7749966	95,2688495	101,865907	77,22664	70,9905882
FR367717	piRNA	TNCGGCTTGTGCTTTAAGT	chr11	103309271	103309300	-1,5199553	7,16E-05	0,00317644	1012,19809	840,236534	1186,75425	934,928949	504,092456	716,514431	863,07135	917,895851
FR364835	piRNA	TTTTATGAAACGGGTGTAA	chr11	5431184	5431212	2,07929478	1,80E-07	7,38E-05	12,333136	7,10059042	8,73530993	4,18499977	15,7239849	11,2225152	11,0323771	12,4544892
FR402585	piRNA	TACAGAAAGGTGACCTCAG	chr11	107857831	107857860	1,69149965	0,00021209	0,00650602	175,306718	127,810628	168,466692	164,888991	129,49164	139,849805	145,118192	150,699319
FR353048	piRNA	TTAAAACTTTGTGTCTTGAT	chr11	103301004	103301029	1,72432054	6,41E-05	0,00299548	59,9038033	55,6212917	71,1303809	64,4489965	68,4455812	58,7023871	74,6807068	72,2360371
FR391061	piRNA	TGTCATTGTGGGTAGCCAT	chr11	5431134	5431163	2,05077478	3,38E-06	0,00052523	108,355409	128,994059	103,575818	94,5809948	104,518252	135,533453	122,204793	85,9359752
FR237942	piRNA	TGTGATATGTGGAATAAAC	chr12	99682063	99682092	-2,3899276	0,00019907	0,00624251	47,5706673	35,5029521	39,9328454	31,8059983	24,0484474	34,5308159	30,5511982	24,9089783
FR254413	piRNA	TGGTGGTTAGATGCCAAAA	chr12	99643079	99643109	-1,8845375	2,20E-06	0,00043236	96,9032112	60,3550186	69,8824795	71,1449961	34,2227906	50,9329535	59,4051077	39,8543653
FR075716	piRNA	TGGTAATAGGTCAGTTAGA	chr12	99655487	99655513	-1,5636672	2,64E-05	0,00177183	27,3090868	35,5029521	22,4622255	20,9249989	38,847492	28,4879231	22,9133987	23,6635294
FR151602	piRNA	TGCAGTGGGCTGCATAGG	chr12	99640618	99640643	1,62776295	5,57E-06	0,00071148	33,4756548	43,7869743	13,7269156	30,1319984	31,4479697	70,7881727	30,5511982	32,3816718
FR304388	piRNA	TGCTAGCAGTTTGGCAGCC	chr12	99685193	99685221	1,6361737	0,00457843	0,04438342	29,9519016	36,6863839	22,4622255	41,0129978	11,0992834	25,0348415	29,7025538	31,1362229
FR171667	piRNA	TAATTTGGCCTTGACATGG	chr12	99650547	99650575	1,51560121	8,42E-06	0,00087134	8,80938283	7,10059042	6,2395071	9,2069995	7,39952229	15,5388672	11,0323771	8,71814241
FR176053	piRNA	TAGCTTTTAACAGAAAGTA	chr12	99625873	99625902	1,83646858	4,87E-06	0,00067204	458,968846	343,195204	308,231651	369,95398	754,751274	491,200857	348,792847	340,007554
FR210227	piRNA	TAAGAATTGAGCCTTATTT	chr12	99642148	99642176	1,53976408	3,26E-06	0,00051397	15,8568891	4,73372695	6,2395071	12,5549993	10,1743432	9,49597438	10,1837327	12,4544892
FR034787	piRNA	TTCCTACAAATTGGCATCC	chr12	99680832	99680859	1,53230054	0,00325081	0,03564797	28,1900251	29,5857934	29,9496341	23,4359987	34,2227906	25,8981119	26,3079763	18,6817337
FR301979	piRNA	TGAGGGCTTCATTTCTCTA	chr12	99655738	99655765	1,63268978	0,0009076	0,01607433	55,4991119	37,8698156	54,9076624	58,5899968	28,6731489	36,2573567	43,2808642	54,7997523
FR353828	piRNA	TAGTTACCTTTGGCTAATT	chr12	99678291	99678313	2,11333637	0,00461894	0,04468388	25,5472102	21,3017713	19,9664227	17,576999	38,847492	29,3511935	19,5188211	19,9271827
FR227678	piRNA	TCTTAATAATAATTAGATG	chr12	99639286	99639314	2,83810158	2,99E-05	0,00191699	101,307903	101,775129	82,3614937	100,439995	162,78949	131,217101	103,534616	90,9177709
FR113856	piRNA	TGAGAGTCAAGTGGCTTGT	chr13	53497240	53497269	-1,7435858	0,0006689	0,01325702	36,9994079	20,1183395	37,4370426	46,0349975	8,32446258	14,6755968	36,491709	31,1362229
FR280787	piRNA	TCTCATAGGTGATGTAAAA	chr13	51239801	51239829	1,74508862	0,00020118	0,00628708	82,8081986	40,2366791	48,6681553	83,6999954	99,8935509	79,4208766	63,6483297	57,2906501
FR273779	piRNA	TGCCTCAACATACAAACCT	chr14	25246870	25246898	-1,9633733	3,12E-05	0,00195663	173,544842	185,798783	200,912128	160,703991	523,516202	372,069542	215,555676	188,062786
FR158031	piRNA	TGATGAATAAAGTCTTCGA	chr14	45560086	45560114	1,50283505	0,00247543	0,02994225	63,4275564	62,7218821	78,6177894	68,6339963	81,3947452	71,6514431	70,4374848	82,1996285
FR232367	piRNA	GTGATCACAGAAATGGACT	chr14	45399503	45399532	1,93992091	0,00012935	0,00463542	11,4521977	15,3846126	17,4706199	20,0879989	11,0992834	13,8123264	13,5783103	18,6817337
FR047640	piRNA	TCTTCTCTTGAGATAGAAC	chr14	24927395	24927424	2,14401254	0,00550049	0,05003547	34,3565931	29,5857934	51,1639582	51,0569972	12,0242237	37,9838975	33,9457758	36,1180186
FR061626	piRNA	AGCTTTGCCCTTCTTAGGG	chr15	74477014	74477034	-2,6928074	1,73E-05	0,00135211	236,09146	197,6331	219,63065	191,67299	274,707265	202,868544	196,036855	174,362848
FR300310	piRNA	CGACACAATAGCCAGATAC	chr15	5663705	5663736	-2,6359385	9,10E-05	0,00370438	88,0938283	110,059152	122,294339	127,223993	92,4940286	75,1045246	108,626483	97,1450154
FR150885	piRNA	TCAAATTTTTGTCAAATTCT	chr15	78495695	78495721	-1,8871846	1,05E-05	0,00099971	118,926668	163,31358	106,071621	147,311992	93,4189689	112,225152	148,512769	128,281238
FR052383	piRNA	TGATTGCCCCCTTAGTTGC	chr15	74471816	74471843	-1,8487595	1,95E-05	0,00147831	27,3090868	17,7514761	26,2059298	23,4359987	4,62470143	13,8123264	17,8215323	24,9089783
FR298080	piRNA	TGCCGTGTGATCTGATGTG	chr15	78497592	78497620	-1,6691318	0,00125282	0,01942335	37,8803462	43,7869743	31,1975355	35,990998	29,5980892	30,2144639	20,3674655	33,6271207
FR001803	piRNA	TGGCAGTCACCCTAAATCT	chr15	59099374	59099402	-1,6376584	0,00265587	0,03126673	36,1184696	20,1183395	21,2143241	17,576999	17,5738654	14,6755968	17,8215323	13,6999381
FR132381	piRNA	TTGTTGACTCAGATGTTTTA	chr15	59107470	59107490	-1,5661124	0,0013163	0,01995487	431,659759	518,343101	565,299343	366,60598	1167,27464	726,873675	644,969741	494,44322
FR007262	piRNA	TTAAAATCAAACCTCAAGG	chr15	59090075	59090104	1,52969976	0,00016643	0,00551736	149,759508	194,082805	182,193607	146,474992	216,436027	205,458355	142,572258	154,435666
FR346759	piRNA	TAAGCAAACTGAATGTGAT	chr15	72934490	72934518	1,85144174	0,00546073	0,04985165	14,9759508	21,3017713	7,48740851	6,69599964	20,3486863	27,6246527	8,48644396	8,71814241
FR302665	piRNA	TAACAGGCTTTAGGCTTTG	chr15	83185733	83185759	1,63390448	8,24E-06	0,00086248	67,8322478	66,2721773	76,1219866	73,655996	81,3947452	61,2921983	56,8591745	49,8179566
FR170307	piRNA	TAGCTTATAACTCTGAATCT	chr15	59082929	59082955	1,50266857	6,14E-08	3,64E-05	25,5472102	41,4201108	23,710127	37,6649979	46,2470143	23,3083008	32,248487	32,3816718
FR229980	piRNA	TAGAAGGTAGTATTTTTCC	chr15	79759626	79759654	1,69952964	0,00360378	0,03805301	95,1413346	97,0414025	107,319522	89,5589951	179,438416	123,447667	112,02106	114,5813
FR204176	piRNA	TTCGTTTCTTTAATGAGGG	chr15	74467519	74467547	1,54356799	5,08E-06	0,00067204	32,5947165	23,6686347	26,2059298	20,9249989	35,1477309	37,1206271	35,6430646	24,9089783
FR378405	piRNA	TAAAATGAGAGACTTTAAA	chr15	59103969	59104000	1,94233836	0,00016211	0,00543733	7,92844455	10,6508856	3,74370426	2,51099986	19,423746	10,3592448	2,54593319	3,73634675
FR014971	piRNA	TGAGAATCAAATTTAACAG	chr15	59089129	59089159	1,63571398	0,0010518	0,01766617	7,92844455	5,91715869	7,48740851	6,69599964	5,54964172	9,49597438	10,1837327	8,71814241
FR293825	piRNA	TAAGATAAATTCTTAGGAT	chr15	78493771	78493801	2,04034141	0,00065605	0,01311449	123,33136	88,7573803	98,5842121	124,712993	86,9443869	67,3350911	96,7454611	69,7451393
FR143411	piRNA	TGGCAGGAAAAAAAAATA	chr17	27451828	27451854	-1,8567634	0,00271172	0,0317314	95,1413346	94,674539	91,0968036	149,822992	68,4455812	82,8739582	94,1995279	99,6359133
FR252303	piRNA	TGAAAAATTTCACAAGTCA	chr17	22655532	22655562	2,30134166	2,59E-05	0,0017429	57,2609884	40,2366791	72,3782823	42,6869977	26,8232683	31,0777343	59,4051077	38,6089164
FR038038	piRNA	TGATAAATTGTAAAATGTC	chr17	27458514	27458540	1,65049852	5,94E-05	0,00287495	43,1659759	41,4201108	34,9412397	56,0789969	30,5230295	25,0348415	36,491709	34,8725696
FR324460	piRNA	TTTAAAGGGAAAAATACCT	chr17	27482929	27482958	1,59511247	6,32E-05	0,00298909	13,2140743	14,2011808	11,2311128	18,413999	21,2736266	10,3592448	18,6701767	14,945387
FR265862	piRNA	TCCTCAGAATAGATTGAGG	chr17	66563098	66563128	2,30845239	0,00088422	0,01580843	44,9278525	72,189336	84,8572965	63,6119965	86,9443869	82,0106878	67,0429073	52,3088545
FR326547	piRNA	TTATAACAAGGCTCTTAAG	chr17	66560231	66560261	1,52056496	2,30E-08	2,03E-05	16,7378274	22,485203	26,2059298	20,0879989	10,1743432	20,7184896	20,3674655	21,1726316
FR233411	piRNA	TGACAAGTGTCTTTTATTTC	chr17	27468478	27468506	1,76502618	0,00372434	0,03895138	64,3084947	49,704133	74,8740851	105,461994	107,293073	70,7881727	84,8644396	112,090402
FR265654	piRNA	TCTCAAGGACTGATAGGAT	chr17	66503170	66503198	1,50558311	5,73E-07	0,0001721	84,5700752	106,508856	69,8824795	77,8409958	121,167178	114,814963	79,7725732	94,6541176
FR292031	piRNA	CAGAACACTTTTGGTTTCA	chr17	66569874	66569901	1,60976048	9,18E-06	0,00091357	830,724801	701,77502	592,753174	777,572958	575,312858	689,753048	670,429073	803,314551
FR039432	piRNA	TACTGAAGGTAACACTCTT	chr17	27447616	27447645	1,67018507	4,30E-10	7,87E-07	152,402323	112,426015	143,508663	191,67299	61,0460589	62,1554687	134,085815	155,681114
FR262810	piRNA	TCCCTGCACACTTGTTAGG	chr17	27482896	27482920	2,30464694	0,00210778	0,02726672	327,709041	323,076864	349,412397	327,266982	337,603205	318,546777	340,306403	313,853127
FR296623	piRNA	TGCCTTATGCTTTCCCTTTC	chr18	67201207	67201236	-1,5575659	0,00378342	0,03941169	211,425188	163,31358	190,928917	198,368989	168,339132	164,021376	194,339567	178,099195
FR065484	piRNA	TCAATGACGCCTGAGCAAT	chr18	67216534	67216563	1,62067354	0,00245459	0,0298083	43,1659759	54,4378599	26,2059298	26,7839985	29,5980892	31,0777343	50,0700193	37,3634675
FR220798	piRNA	TAGTGACTGGTTAAGCTCG	chr18	67218268	67218293	1,5384295	2,50E-06	0,00045534	8,80938283	11,8343174	6,2395071	10,0439995	14,7990446	21,58176	12,7296659	13,6999381
FR019473	piRNA	TTCTTTGTACTGTCAGTTTG	chr18	67198516	67198543	1,98263803	0,00393427	0,04027625	50,2134822	50,8875647	66,1387752	45,1979975	56,4213575	64,7452799	52,6159525	57,2906501
FR000440	piRNA	TGTCATTGCGTGAACACAG	chr18	67218897	67218928	1,83686555	2,53E-10	6,21E-07	38,7612845	31,9526569	33,6933383	61,9379966	58,271238	88,916851	41,5835754	38,6089164
FR336236	piRNA	TTGAAGAAGGCTGAAGTTC	chr18	67198315	67198342	1,52287583	2,02E-05	0,00151318	330,351856	247,337233	175,9541	229,337988	121,167178	119,994585	236,771786	173,117399
FR266341	piRNA	TAAACATACAGATGAAACA	chr18	67224786	67224814	1,64202863	3,04E-05	0,00192651	409,636302	268,639004	304,487946	436,076976	396,799383	426,455577	407,34931	429,679876
FR344015	piRNA	ATAAACATCAATGTTTATG	chr18	67220040	67220070	1,69074218	0,00140385	0,02085057	64,3084947	53,2544282	57,4034653	51,8939972	47,1719546	47,4798719	61,9510409	37,3634675
FR290232	piRNA	TACATAAAAAGGCATCCTT	chr19	37332285	37332313	1,53201242	5,82E-06	0,00072049	51,9753587	27,21893	38,684944	36,827998	22,1985669	31,9410047	36,491709	37,3634675
FR143280	piRNA	TGTGAGATAAAGATGATTA	chr2	92431005	92431028	-2,1955953	1,81E-21	3,31E-17	267,805238	239,053211	177,202002	250,262986	181,288296	177,833702	198,582789	215,462662
FR110851	piRNA	TGTGAGTTAAATGAGCCCA	chr2	92420244	92420273	-1,7260112	8,03E-06	0,00085955	227,282077	229,585757	258,315594	199,205989	261,758101	303,00791	208,766521	201,762724
FR072314	piRNA	TGCCTCCTTAGCTTTAGGG	chr2	92399713	92399741	-1,6868991	2,51E-06	0,00045534	161,211706	179,881624	184,68941	210,923989	197,937221	195,09911	208,766521	224,180805
FR371739	piRNA	TGGGACAGGAACCTCAAA	chr2	151085151	151085179	-1,6841121	1,20E-05	0,00107161	95,1413346	98,2248342	128,533846	61,9379966	158,164789	106,182259	95,8968167	125,790341
FR150353	piRNA	TACCATTTGCTTAAACAGC	chr2	150976021	150976050	1,77314711	0,00081654	0,01505873	59,022865	54,4378599	49,9160568	52,7309971	86,9443869	75,1045246	79,7725732	54,7997523
FR306915	piRNA	TATTTAATTTATGAGTGCCC	chr2	92422594	92422625	1,5711695	0,00010543	0,00404166	15,8568891	11,8343174	18,7185213	12,5549993	15,7239849	19,8552192	12,7296659	11,2090402
FR400487	piRNA	TACCTGGGATCTTTTCTGTT	chr2	92403919	92403946	1,51807833	1,72E-05	0,00135211	165,616397	201,183395	188,433114	187,48799	177,588535	145,892697	205,371944	189,308235
FR399018	piRNA	TTTGAAACATTTGTACTCAC	chr2	151236856	151236884	1,77382467	5,75E-06	0,00072049	89,8557049	124,260332	91,0968036	104,624994	75,8451035	85,4637694	131,539881	100,881362
FR300823	piRNA	TACATGTTCTGTTACCATG	chr2	151155224	151155253	1,62294731	1,05E-08	1,28E-05	278,376498	282,840185	195,920523	140,615992	427,322412	303,00791	167,182946	245,353436
FR370290	piRNA	TTTCAGATCTTATGGTAAC	chr2	92395550	92395580	1,64129306	9,44E-06	0,00092563	155,926076	253,254392	182,193607	148,148992	320,954279	265,024012	157,847858	159,417461
FR122619	piRNA	TAGAAAATAAAACCAACGT	chr2	92386632	92386661	1,63572332	2,28E-06	0,00044246	474,825735	708,875611	691,337386	532,331971	741,80211	823,55996	680,612805	574,15195
FR113178	piRNA	TAGGGTGACCGAGTAAGA	chr2	92386424	92386450	1,51200837	4,93E-07	0,00016111	82,8081986	138,461513	129,781748	101,276994	115,617536	153,662131	113,718349	127,035789
FR214649	piRNA	TATCAGTTACAAAAACCAC	chr2	92432476	92432507	1,56544586	6,67E-05	0,00304385	61,6656798	50,8875647	31,1975355	63,6119965	39,7724323	41,4369791	50,0700193	48,5725077
FR283982	piRNA	TAAATGATTTTGTCTATAGT	chr2	151233833	151233863	1,72331032	0,00138884	0,02069466	36,1184696	57,9881551	32,4454369	32,6429982	49,9467755	50,9329535	39,0376422	54,7997523
FR376528	piRNA	TCCAGTCAGATGAACTGAA	chr2	127518060	127518090	1,81190165	5,74E-08	3,64E-05	106,593532	57,9881551	112,311128	100,439995	138,741043	67,3350911	108,626483	99,6359133
FR264318	piRNA	TGAAGGTCAAAGTTGTGTC	chr2	92407543	92407568	1,81949302	0,00243473	0,02964002	300,399955	190,53251	273,290411	366,60598	228,460251	161,431564	305,511982	316,344025
FR163185	piRNA	TCAATTCTAAGGAATTTCA	chr2	151146189	151146218	1,82419164	2,00E-05	0,00150915	551,467365	557,396348	566,547244	568,322969	474,494367	637,956824	589,807855	670,051517
FR195614	piRNA	TATAACAGTTTGTTTTATAT	chr2	151085479	151085507	1,83720914	3,35E-05	0,0020382	104,831656	93,4911072	144,756565	123,038993	175,738654	159,705024	172,274812	141,981176
FR394595	piRNA	GAGGGTTTTCTTTATCCTTT	chr2	151146895	151146924	2,01224367	5,33E-07	0,00016538	17,6187657	10,6508856	6,2395071	13,3919993	38,847492	24,1715712	17,8215323	9,96359133
FR115346	piRNA	TGACAAACTAAATGGTCAT	chr2	151239341	151239368	2,02778346	7,46E-06	0,00082791	44,9278525	61,5384503	71,1303809	61,1009967	32,37291	61,2921983	43,2808642	62,2724458
FR404042	piRNA	TAACATTGGGCCTGTTGGT	chr2	92417087	92417116	2,2258559	0,00476586	0,04548055	248,424596	189,349078	233,357565	235,196987	336,678264	245,168793	219,798898	193,044582
FR302832	piRNA	TGAGTGATAAGATTGAGTA	chr3	20387748	20387776	-2,4567649	0,00032997	0,00857129	722,369392	586,982142	640,173428	828,629955	302,455474	428,182118	593,202433	668,806068
FR120499	piRNA	TACCTTTTCCTAATGTTAAA	chr3	20365184	20365213	-1,6582085	0,00242085	0,02955537	278,376498	248,520665	333,189679	312,200983	327,428861	284,015961	328,425381	340,007554
FR387206	piRNA	TAGAAAGAAATTCTGCAGT	chr3	124117322	124117351	1,66128019	0,00471862	0,04520981	246,662719	214,201144	197,168424	236,870987	160,93961	191,646028	182,458545	266,526068
FR116914	piRNA	TCTGCAGGCACCTTCTCTG	chr4	42602194	42602223	-2,2835224	0,00086627	0,01564505	23,7853337	43,7869743	29,9496341	28,4579984	66,5957006	50,0696831	39,0376422	48,5725077
FR199585	piRNA	TGGTGACTGTCTGGGATGA	chr4	93986746	93986769	-1,9746459	0,00211607	0,02732837	12,333136	22,485203	16,2227184	13,3919993	24,9733877	25,0348415	12,7296659	17,4362848
FR211852	piRNA	TGGGACAGTCCAAAATGAT	chr4	93992137	93992162	-1,763789	5,07E-05	0,00257718	997,222137	817,751331	1121,86338	1212,81293	188,687818	391,924761	803,666243	1010,05907
FR150017	piRNA	TGCCAAGGAAGTTTTTACT	chr4	42290636	42290664	-1,6780759	0,00109764	0,018075	156,807014	108,87572	104,823719	157,355991	30,5230295	74,2412542	121,356149	119,563096
FR126707	piRNA	TCCAGTCCCATAGACCTCA	chr4	57372554	57372583	-1,6341981	0,00023603	0,00700544	41,4040993	33,1360886	33,6933383	51,8939972	25,898328	29,3511935	39,8862866	31,1362229
FR213523	piRNA	TAGACCAAAATTTTCATCTC	chr4	42421076	42421106	-1,5884722	0,00021985	0,00664389	244,019904	188,165646	212,143241	190,83599	343,152846	247,758604	208,766521	205,499071
FR341779	piRNA	TGTAACATGACCATAAAAT	chr4	123514115	123514145	1,53677173	0,00242495	0,02957504	61,6656798	56,8047234	72,3782823	65,2859964	61,9709992	43,1635199	61,9510409	73,481486
FR276285	piRNA	TGAAATAGTGTTTCGATGT	chr4	93949314	93949343	1,52743522	0,00010083	0,00393693	29,0709634	44,970406	38,684944	32,6429982	42,5472532	65,6085503	28,8539095	36,1180186
FR305661	piRNA	TGAAAAGTTATGGGATGCT	chr4	61889403	61889430	1,77158568	0,0025703	0,03065474	377,922524	275,739595	340,677087	398,411978	75,8451035	180,423513	246,106875	315,098576

FR117833	piRNA	TGAAATTTTATTTTACTTTC	chr4	93960636	93960665	1,65409225	1,50E-05	0,00123349	77,5225689	112,426015	82,3614937	81,1889956	99,8935509	101,002637	100,140039	99,6359133
FR003397	piRNA	TACACGGAGCTTAGGAAGT	chr4	123572278	123572308	1,54456621	0,00498589	0,04704102	512,706081	452,070924	424,286482	616,868966	295,980892	338,401996	484,57595	368,652879
FR073542	piRNA	TGAAAGGATATATGGCTGT	chr4	93994476	93994503	1,95100341	0,00023887	0,00704873	4261,09848	4110,05842	3671,32597	3953,15078	4741,24391	4539,93902	4053,12563	3926,90043
FR017976	piRNA	TGAACTTTCTGTTGGTCAA	chr4	93998075	93998101	2,26022225	5,10E-05	0,00258419	86,3319518	68,6390408	66,1387752	69,4709962	29,5980892	44,8900607	78,9239288	72,2360371
FR204403	piRNA	TAAAACTCGCTCTCGTGTT	chr4	123515094	123515122	2,65999798	0,00524801	0,04858791	72,2369392	75,7396312	73,6261837	86,2109953	85,0945063	71,6514431	72,983418	74,726935
FR261079	piRNA	TGGAGCTCAGAGAGAAGC	chr5	150627666	150627686	-1,7171982	1,32E-05	0,00113806	19,3806422	15,3846126	19,9664227	19,250999	12,0242237	24,1715712	22,0647543	27,3998762
FR048394	piRNA	TGAGTTATTGCCTTCTCTG	chr5	113767220	113767247	-1,6407994	9,63E-06	0,00093813	233,448645	213,017713	299,496341	227,663988	261,758101	271,066905	213,858388	254,071579
FR266385	piRNA	TACTGCTGAGTGAGCCACA	chr5	113784228	113784251	-1,5921705	0,00153284	0,02208574	35,2375313	22,485203	9,98321135	31,8059983	24,0484474	23,3083008	33,0971314	16,1908359
FR384675	piRNA	TGCCGTCGGTGCTAGCAG	chr5	150626146	150626176	1,51862795	0,00076396	0,01443283	69,5941244	50,8875647	41,1807468	51,8939972	48,0968949	61,2921983	50,9186637	63,5178947
FR253835	piRNA	TGAAAACTGTGTCTGTTTC	chr5	150616042	150616067	1,7347859	2,93E-06	0,00049242	206,139558	321,893433	304,487946	191,67299	453,22074	520,55205	359,825224	333,780309
FR366488	piRNA	TGATAAGTTATAATTATCT	chr5	113761250	113761278	1,74100916	0,00090095	0,01600307	28,1900251	42,6035425	31,1975355	40,1759978	21,2736266	34,5308159	42,4322198	47,3270588
FR368487	piRNA	ACAAAAACCATGCAACGCT	chr5	150614701	150614729	1,73320489	8,58E-07	0,00021529	41,4040993	20,1183395	29,9496341	34,3169981	49,9467755	40,5737087	25,4593319	31,1362229
FR279105	piRNA	TATCTATGTAGTTATACAG	chr5	150610004	150610033	1,78662377	7,03E-05	0,00313064	278,376498	351,479226	223,374354	272,024985	126,716819	183,876595	266,47434	219,199009
FR397568	piRNA	TGGATTGTACAAAGAGTCA	chr5	150603701	150603731	1,57214033	0,00311688	0,03470109	290,709634	439,053175	460,475624	262,817986	716,828722	541,27054	405,652021	342,498452
FR082597	piRNA	TTTTAAAAAAAAATCTGTGG	chr5	150619955	150619982	2,19115805	0,00049	0,01104423	7,04750627	8,28402216	7,48740851	6,69599964	7,39952229	6,90616319	9,33508835	7,4726935
FR330401	piRNA	TTTGAACATGTGTCTCACT	chr5	130629699	130629727	2,85577798	0,00011705	0,004301	141,831064	123,076901	72,3782823	92,069995	110,992834	94,0964734	91,6535947	107,108607
FR163800	piRNA	TCCTACTGAAGAGGCTTCA	chr6	85938104	85938133	-1,5672407	0,00446272	0,04366972	21,1425188	8,28402216	6,2395071	11,7179994	36,9976115	16,4021376	36,491709	26,1544272
FR080742	piRNA	TATTTCCAAAAGTCTGGTG	chr6	127764933	127764963	1,52905857	0,00026103	0,00749267	196,449237	196,449668	195,920523	218,456988	140,590924	196,825651	206,220588	222,935356
FR386772	piRNA	TGAAAATGAAGCAGTTCCA	chr6	127738885	127738914	1,58033596	0,00110124	0,018075	7,04750627	7,10059042	11,2311128	8,36999954	12,0242237	7,76943358	11,0323771	13,6999381
FR322734	piRNA	TAAAAACTAATGTTACTTT	chr6	128140775	128140803	1,77391627	6,59E-05	0,00303472	71,356001	72,189336	71,1303809	70,3079962	56,4213575	64,7452799	64,4969741	49,8179566
FR045462	piRNA	TATTTGTTGTCTCCAAATCT	chr6	128397014	128397044	1,78988336	0,00044645	0,01040185	7,04750627	10,6508856	7,48740851	5,85899968	23,1235072	6,90616319	25,4593319	6,22724458
FR024427	piRNA	TAGGGCTTAAGAAGTTATT	chr6	127768547	127768575	1,54610721	0,00088185	0,01578766	59,9038033	65,0887456	84,8572965	64,4489965	122,092118	75,967795	86,5617284	84,6905263
FR167946	piRNA	TCCGTAAGGGTGAATCCAT	chr6	127768408	127768437	2,86163195	0,00057597	0,01208819	22,9043954	16,5680443	9,98321135	22,5989988	10,1743432	12,949056	15,2755991	19,9271827
FR330918	piRNA	TGAAAAAGGCCTACCTTGA	chr6	127785030	127785058	3,28192866	0,002335	0,02897072	15,8568891	11,8343174	14,974817	12,5549993	19,423746	17,265408	19,5188211	16,1908359
FR228320	piRNA	GGAAGGCACAGAATCTGT	chr7	80266474	80266502	-1,8025411	1,28E-10	3,89E-07	26,4281485	28,4023617	21,2143241	38,5019979	23,1235072	15,5388672	22,0647543	13,6999381
FR261537	piRNA	TGGATGTGAGCGCCTGGA	chr7	80284272	80284298	-1,6381334	0,00096376	0,01676103	477,46855	408,283949	495,416863	468,719974	564,213575	566,305381	474,392217	393,561857
FR056969	piRNA	TGAAAAACAGCTCTGCCAC	chr7	77077671	77077698	1,52179475	0,00019907	0,00624251	20,2615805	21,3017713	18,7185213	17,576999	19,423746	24,1715712	11,8810215	12,4544892
FR222227	piRNA	TAAGTGAGTTGCTTAAAGC	chr7	80251632	80251660	1,57203505	0,00232091	0,02887422	289,828695	244,97037	249,580284	248,588986	199,787102	185,603136	250,350097	235,389845
FR075803	piRNA	TGCTGATAGAGGTACCTAT	chr7	132473425	132473453	1,52407085	7,20E-05	0,00317797	303,042769	205,917122	212,143241	292,112984	104,518252	134,670182	232,528564	205,499071
FR061515	piRNA	TATTTACATGGATCAGAGA	chr7	77032253	77032281	2,80583152	0,00020996	0,00645121	10,5712594	8,28402216	12,4790142	6,69599964	17,5738654	5,17962239	6,78915516	8,71814241
FR193085	piRNA	TGCCAATATATGGGTCACT	chr7	77079651	77079679	1,57152064	0,00032402	0,00850443	156,807014	142,011808	179,697804	131,408993	67,5206409	91,5066622	151,058702	181,835542
FR200839	piRNA	TGGTATCTACATATCTATG	chr7	132473016	132473045	1,70516266	5,37E-05	0,0026746	50,2134822	36,6863839	38,684944	39,3389979	46,2470143	39,7104383	45,8267974	33,6271207
FR291664	piRNA	TATTAGAGCAAGGGACTTG	chr7	80267592	80267620	1,55866556	2,98E-06	0,00049681	51,0944204	42,6035425	52,4118596	57,7529969	37,9225517	43,1635199	50,0700193	51,0634056
FR249856	piRNA	TACTTTTATTCAGGTGTGTC	chr7	60167576	60167603	1,62583526	0,00225895	0,02835372	32,5947165	28,4023617	37,4370426	25,9469986	11,0992834	24,1715712	23,7620431	22,4180805
FR054588	piRNA	TATAAACTTAAATATATACT	chr7	80270455	80270485	1,65529113	0,00075972	0,01437486	28,1900251	18,9349078	24,9580284	12,5549993	26,8232683	27,6246527	25,4593319	32,3816718
FR218632	piRNA	TAACACGTGAAGGTAAACC	chr7	80289514	80289542	1,61266666	0,00064521	0,01301301	73,9988158	101,775129	49,9160568	73,655996	52,7215963	41,4369791	55,1618857	63,5178947
FR083597	piRNA	TGCAAAACTTGCAGGTGTT	chr7	80960335	80960364	1,51177455	0,00195029	0,02627274	61,6656798	65,0887456	49,9160568	51,8939972	65,6707603	62,1554687	56,0105301	44,836161
FR055834	piRNA	TTGTGTTCTGAATCAAGTG	chr7	77048832	77048857	1,534651	6,08E-05	0,00291633	621,06149	549,112326	550,324526	616,031966	470,794606	467,892556	378,4954	592,833684
FR024255	piRNA	TAAACTTTTAAAGTCAGAA	chr7	77049250	77049281	1,62116474	3,73E-05	0,00217476	11,4521977	13,0177491	14,974817	9,2069995	14,7990446	12,949056	16,1242435	8,71814241
FR231465	piRNA	TACTAGGTTTGGAAGGTTT	chr7	80951882	80951911	1,62748798	0,00435334	0,04288469	125,974175	113,609447	136,021255	120,527993	62,8959395	76,8310654	96,7454611	104,617709
FR236096	piRNA	TCAAACATAACCTTGGGTA	chr7	77075454	77075480	1,56108945	0,00018364	0,00585876	28,1900251	48,5207012	28,7017326	30,9689983	17,5738654	29,3511935	30,5511982	32,3816718
FR114359	piRNA	TCATTTAGTGTTGTCAGCTT	chr7	77058421	77058450	1,60826799	5,81E-06	0,00072049	321,542473	459,171514	334,43758	318,896983	422,697711	329,769292	325,879448	295,171393
FR164570	piRNA	TAAATGTCCACTTGGGGAC	chr7	80970104	80970131	1,50907875	8,38E-06	0,00087134	8,80938283	8,28402216	8,73530993	11,7179994	11,0992834	8,63270398	10,1837327	13,6999381
FR337575	piRNA	TGGAAAAACTTGGAGGCG	chr7	80933818	80933844	1,57980721	0,00291997	0,03323393	3381,04113	6796,44847	4758,24811	3652,6678	8988,5697	6812,92998	4349,30253	3854,66439
FR116503	piRNA	TGAAAGGAGTGCCTTTTAG	chr7	77059647	77059675	1,69815084	0,00143453	0,02123825	11,4521977	14,2011808	13,7269156	16,7399991	9,24940286	11,2225152	11,0323771	11,2090402
FR048705	piRNA	TGTAATGCAAAGGTGACCC	chr7	80935614	80935632	1,70893822	0,00225508	0,028344	7,04750627	13,0177491	3,74370426	13,3919993	13,8741043	10,3592448	11,8810215	3,73634675
FR239455	piRNA	TAAAGAGACTTAGGGCAAT	chr7	77049597	77049626	1,75271906	5,33E-08	3,61E-05	30,8328399	98,2248342	18,7185213	25,1099986	39,7724323	42,3002495	31,3998426	24,9089783
FR389525	piRNA	TGAGTAACAGTCTTCAGCC	chr7	80953249	80953277	1,81649466	0,00236579	0,02919461	329,470918	369,230702	117,302733	271,187985	281,181847	336,675455	271,566207	254,071579
FR114986	piRNA	TACAAGGTGTACAGTGTAT	chr7	132476105	132476133	1,82822814	0,00044805	0,01041269	14,9759508	9,4674539	13,7269156	14,2289992	7,39952229	12,949056	15,2755991	16,1908359
FR094852	piRNA	TATTAAAATGTAGCCTTCT	chr7	80934661	80934690	1,86257392	0,00035593	0,0090278	9,69032112	13,0177491	9,98321135	12,5549993	21,2736266	18,1286784	15,2755991	16,1908359
FR008425	piRNA	AAGGAAGAAACCTTCCAG	chr7	77077174	77077203	1,94046491	4,41E-07	0,00014687	713,56001	737,277972	544,085019	631,934966	399,574204	529,184754	494,759683	646,387987
FR000263	piRNA	TAATTGGACCAGAGGCGA	chr7	80283413	80283442	1,9977031	0,00344584	0,03694594	33,4756548	86,3905168	23,710127	41,8499977	58,271238	48,3431423	34,7944202	36,1180186
FR171485	piRNA	TACAATTTAAAGTTTTATTT	chr7	80936708	80936737	2,31888384	0,00073929	0,01411746	1,76187657	7,10059042	12,4790142	4,18499977	10,1743432	11,2225152	5,09186637	3,73634675
FR379582	piRNA	TGGGAATCCGCAGAGGAA	chr8	94716382	94716407	-1,7473255	0,00013175	0,00468934	61,6656798	65,0887456	64,8908738	73,655996	75,8451035	82,8739582	61,9510409	44,836161
FR211700	piRNA	TAGAGTCTACATTGTTCCC	chr8	112646673	112646697	2,87286472	0,00274091	0,03186937	57,2609884	35,5029521	34,9412397	62,7749966	29,5980892	36,2573567	36,491709	54,7997523
FR139110	piRNA	TTATAGGGATGCCTCCGCG	chr8	94714942	94714964	1,69742319	0,00264229	0,03123255	707,393442	562,130075	529,110202	662,903964	350,552369	546,450162	558,408012	398,543653
FR038517	piRNA	TAGCAAGTTGTAAACAAGT	chr8	94716889	94716919	1,51442054	3,14E-10	6,39E-07	14,0950125	17,7514761	12,4790142	11,7179994	7,39952229	11,2225152	7,63779956	4,98179566
FR083423	piRNA	TATTAGATTTGAGGCTTTTT	chr8	28405612	28405639	2,29102507	0,00030795	0,00819705	4,40469142	7,10059042	6,2395071	9,2069995	5,54964172	5,17962239	4,24322198	7,4726935
FR199032	piRNA	TAGAAAGGTTCTTGTGACT	chr8	38156996	38157023	2,74780259	0,00075984	0,01437486	214,068003	275,739595	249,580284	274,535985	118,392357	238,26263	227,436698	235,389845
FR192098	piRNA	TGGCTGGCTGCCTTAACAC	chr9	54097058	54097087	-2,3376985	0,00022896	0,00688489	48,4516056	48,5207012	33,6933383	32,6429982	27,7482086	29,3511935	42,4322198	37,3634675
FR076599	piRNA	TGGGAAAGGAAGTCAAGA	chr9	54070163	54070192	-1,7936033	7,34E-19	6,72E-15	6,16656798	1,18343174	3,74370426	1,67399991	9,24940286	5,17962239	10,1837327	2,49089783
FR075276	piRNA	TAGGAGCTGGGCTGGAGG	chr9	54074164	54074192	-1,5643148	0,00225728	0,02835226	45,8087907	29,5857934	29,9496341	22,5989988	36,0726712	62,1554687	23,7620431	27,3998762
FR212198	piRNA	TATGAGATTGAGGAACAA	chr9	54069231	54069254	4,10301069	0,00499429	0,04709601	494,206377	481,656717	562,80354	509,732972	151,690207	254,664767	385,284556	387,334613
FR391179	piRNA	TGATTCAAATCTCACGAGG	chr9	67571191	67571220	1,59775337	1,44E-05	0,00120814	40,523161	49,704133	44,9244511	55,241997	34,2227906	22,4450304	47,5240862	39,8543653
FR065420	piRNA	TAAATAAAACATCGAAGAC	chr9	67562906	67562934	1,8109904	1,60E-05	0,00130457	207,020497	195,266237	209,647438	185,81399	114,692596	175,243891	191,793633	165,644706
FR105686	piRNA	TAAAGTTTTATTGGAAGAC	chr9	67560458	67560483	1,58267292	0,00210266	0,02726672	71,356001	54,4378599	57,4034653	50,2199973	51,796656	63,8820095	61,9510409	33,6271207
FR218158	piRNA	TCCTCAAGATGGAGTCCAA	chr9	54063865	54063892	2,118525	8,45E-13	5,16E-09	11,4521977	10,6508856	17,4706199	15,0659992	55,4964172	12,949056	10,1837327	4,98179566
FR069467	piRNA	TTACATTTCAGTGAAAAGT	chr9	43956198	43956226	1,50314927	6,71E-06	0,00078346	4,40469142	10,6508856	4,99160568	4,18499977	12,0242237	5,17962239	4,24322198	2,49089783
FR082894	piRNA	CACTTATGGCGCCTCACAC	chr9	54097173	54097201	1,50424538	1,94E-06	0,00040323	1970,65894	3196,44912	2126,42402	1876,5539	2450,16682	2223,78455	1637,03504	1604,1382
FR260035	piRNA	TAATATAAAACTGAAGTTG	chr9	67596095	67596125	1,85776612	0,00033549	0,00865334	3,52375313	3,55029521	9,98321135	6,69599964	14,7990446	20,7184896	11,0323771	9,96359133
FR109626	piRNA	TCCAAACTCTGTGTCAGTG	chr9	67548872	67548900	1,50327833	0,00347957	0,03724215	9,69032112	29,5857934	3,74370426	5,02199973	12,949164	10,3592448	5,94051077	3,73634675
FR040011	piRNA	TCTGAGAACTTATTCAGTG	chr9	67565283	67565311	1,60338275	4,03E-06	0,00061969	665,108404	1026,03532	713,799612	574,181969	1715,76423	1017,7958	778,206911	646,387987
FR031566	piRNA	TGTTTCTCAGTTGACCTGTA	chr9	54078745	54078773	1,66375336	1,59E-06	0,00035896	196,449237	231,952621	237,10127	248,588986	290,43125	220,997222	220,647543	232,898947
FR150813	piRNA	TGATGCTCGTCCTAGCTAT	chr9	67583866	67583895	1,71059915	2,58E-05	0,0017429	151,521385	285,207049	284,521524	207,575989	480,968949	369,47973	263,928407	246,598885
FR299470	piRNA	TGAATTTGAAGTTGTTTCA	chr9	67577016	67577041	1,73232104	0,00496131	0,04689863	284,543066	246,153801	308,231651	315,548983	172,963834	149,345779	219,798898	288,944149
FR079983	piRNA	TACGTCATGAGTAGATCAA	chr9	67547549	67547577	1,74769286	0,00042784	0,0101568	3,52375313	11,8343174	12,4790142	6,69599964	22,1985669	13,8123264	5,94051077	11,2090402
FR310321	piRNA	TGCCTGAACTTTAAGGCCT	chr9	67546881	67546907	1,7672961	0,00018346	0,00585876	47,5706673	33,1360886	33,6933383	26,7839985	47,1719546	69,9249023	26,3079763	34,8725696
FR066718	piRNA	TGGATGGAGGTCTAGGTT	chr9	54068047	54068075	2,32286703	0,00447789	0,04378199	7,04750627	10,6508856	4,99160568	9,2069995	1,84988057	1,7265408	11,0323771	1,24544892
FR361622	piRNA	TATTCCTCAGTGTGTCATA	chr9	54099702	54099730	2,45900032	0,0011497	0,01848502	66,0703713	118,343174	66,1387752	36,827998	61,9709992	95,8230142	46,6754418	58,536099
Name	Type	Seq	Chromosome	Start	End	log2FoldChar	pval	padj	53,7372353	49,704133	38,684944	31,8059983	63,8208798	87,1903102	37,3403534	48,5725077

Table S4: Mapping of the deregulated piRNAs to known piRNA clusters

Chromosome localization	Start	End	Cluster number	Number of deregulated piRNA/cluster
chr1	57348009	57362762	9	1
chr1	91551282	91560929	22	1
chr1	92988215	93000921	23	2
chr10	85848012	85855029	88	1
chr10	94670068	94680641	93	1
chr10	127690863	127704362	96	1
chr10	62651034	62694021	69	2
chr10	86560003	86596028	89	2
chr10	85740002	85774027	86	3
chr10	66680002	66709815	70	4
chr11	107990084	108001964	167	1
chr11	5526240	5536022	103	2
chr11	103420011	103453008	162	2
chr12	98379001	98455979	204	13
chr13	53393064	53407849	226	1
chr14	24078000	24124942	248	1
chr14	24421041	24433017	250	1
chr14	44759007	44791890	255	1
chr14	44932114	44946021	256	1
chr15	73094010	73110057	276	1
chr15	79924089	79934896	287	1
chr15	83349097	83369988	299	1
chr15	74625035	74667908	279	3
chr15	78642001	78675022	283	3
chr15	59245061	59304022	275	6
chr17	22511012	22522028	340	1
chr17	66148680	66159006	372	1
chr17	66164005	66233002	373	3
chr17	27283000	27370953	350	6
chr18	67028034	67088022	389	8
chr19	37252052	37264978	403	1
chr2	127687043	127706875	443	1
chr2	151139086	151162029	456	1
chr2	151245426	151267020	457	2
chr2	151315256	151336029	458	3
chr2	151398016	151420600	459	3
chr2	92536004	92613016	430	11
chr3	20460000	20469958	494	1
chr3	124398138	124419933	520	1
chr4	57353369	57369005	535	1
chr4	62216001	62243018	538	1
chr4	123888001	123899426	556	1
chr4	123831081	123846028	555	2

chr4	94277004	94338846	540	6
chr5	130149032	130159006	633	1
chr5	113316068	113373001	614	3
chr5	149786048	149834611	657	7
chr6	85983003	85994796	685	1
chr6	128179025	128204765	709	1
chr6	128440015	128453028	710	1
chr6	127778004	127842026	708	5
chr7	52903534	52918016	737	1
chr7	125323006	125337723	758	3
chr7	73098005	73154023	740	7
chr7	73774005	73842029	741	8
chr7	69879006	69940023	739	10
chr8	27291000	27301030	773	1
chr8	37089005	37096684	775	1
chr8	110118003	110134528	803	1
chr8	92185128	92202847	794	3
chr9	44139170	44153876	832	1
chr9	54206007	54266025	840	9
chr9	67689001	67763015	849	10

97% deregulated piRNAs were mapped to known clusters using the piRNA cluster data base provided on the web site http://www.smallrnagroup.uni-mainz.de [1,2]. Only piRNA clusters with deregulated piRNA are listed.
